# Patient, carer, and staff perceptions of robotics in motor rehabilitation: a systematic review and qualitative meta-synthesis

**DOI:** 10.1186/s12984-021-00976-3

**Published:** 2021-12-25

**Authors:** Despina Laparidou, Ffion Curtis, Joseph Akanuwe, Khaled Goher, A. Niroshan Siriwardena, Ayse Kucukyilmaz

**Affiliations:** 1 Community and Health Research Unit, School of Health and Social Care, University of Lincoln, Brayford Pool, Lincolnshire, Lincoln, LN6 7TS UK; 2 Diabetes Research Centre, College of Medicine, Biological Sciences and Psychology, Leicester General Hospital, Gwendolen Road, Leicester, LE5 4PW UK; 3 School of Engineering, University of Lincoln, Brayford Pool, Lincolnshire, Lincoln, LN6 7DQ UK; 4 School of Computer Science, University of Nottingham, Jubilee Campus, Wollaton Road, Nottingham, NG8 2DU UK

**Keywords:** Robotics, Motor rehabilitation, Patients, Carers, Staff, Perceptions, Experiences, Systematic review, Meta-synthesis

## Abstract

**Background:**

In recent years, robotic rehabilitation devices have often been used for motor training. However, to date, no systematic reviews of qualitative studies exploring the end-user experiences of robotic devices in motor rehabilitation have been published. The aim of this study was to review end-users’ (patients, carers and healthcare professionals) experiences with robotic devices in motor rehabilitation, by conducting a systematic review and thematic meta-synthesis of qualitative studies concerning the users’ experiences with such robotic devices.

**Methods:**

Qualitative studies and mixed-methods studies with a qualitative element were eligible for inclusion. Nine electronic databases were searched from inception to August 2020, supplemented with internet searches and forward and backward citation tracking from the included studies and review articles. Data were synthesised thematically following the Thomas and Harden approach. The CASP Qualitative Checklist was used to assess the quality of the included studies of this review.

**Results:**

The search strategy identified a total of 13,556 citations and after removing duplicates and excluding citations based on title and abstract, and full text screening, 30 studies were included. All studies were considered of acceptable quality. We developed six analytical themes: logistic barriers; technological challenges; appeal and engagement; supportive interactions and relationships; benefits for physical, psychological, and social function(ing); and expanding and sustaining therapeutic options.

**Conclusions:**

Despite experiencing technological and logistic challenges, participants found robotic devices acceptable, useful and beneficial (physically, psychologically, and socially), as well as fun and interesting. Having supportive relationships with significant others and positive therapeutic relationships with healthcare staff were considered the foundation for successful rehabilitation and recovery.

**Supplementary Information:**

The online version contains supplementary material available at 10.1186/s12984-021-00976-3.

## Background

Mobility difficulties can often occur after accidents, injuries, or following illness. Lack of mobility or difficulties in mobility has been linked to decreased quality of life [1], poor psychological health, such as depression [2, 3], restrictions in social life [4, 5], increased falls, healthcare utilization and expenditures, and decreased compliance with recommended preventive services [6].

Motor rehabilitation is important for re-establishing or improving patients’ mobility and functionality and has been proven highly beneficial, for example, in studies with stroke patients [7, 8]. In recent years, rapid technological developments have led to the design of technology-supported motor training that can help support more traditional physiotherapy, providing the means to increase the intensity and repetition of task-specific treatments and, therefore, facilitate recovery. Robotic rehabilitation devices, in particular, have often been used successfully for motor training, for example improving upper [9–11] and lower extremity movements [12], as well as walking and gait pattern functions [13, 14], in a variety of conditions, such as cerebral palsy [12, 13] or stroke [9–11, 14]. Systematic reviews exploring the effectiveness of robotic rehabilitation devices for people in stroke recovery, have also shown that such devices can be beneficial for upper limb [15, 16] and gait rehabilitation [17], as well as being cost-effective [18]. However, not all studies have reported positive findings. A randomised controlled trial, comparing the clinical effectiveness of robotic training with an enhanced upper limb therapy programme (based on repetitive functional task practice) and with usual care, did not support the use of robotic training in routine clinical practice [19]. The authors of the study noted various reasons for why the improvements in impairment did not translate into improved function, such as not providing sufficient guidance to participants about making the “best use of any reduction in impairment in day-to-day activities”, not incorporating goal-orientated repetitive functional task practice in their programme (like the second arm of the study did, which resulted in more positive results), or recruiting participants who had little prospect of recovery [19].

Quantitative studies have also shown that robotic devices aimed at motor rehabilitation are well accepted by both patients and therapists [20–24] and are considered beneficial and enjoyable [22–24]. Qualitative studies have described how patients felt that using an exoskeleton had physical, social and psychological benefits, such as enhanced independence and activities of daily living (ADLs), improved mood, as well as increased energy and possibilities to interact with others [25, 26]. Therapists also found the robotic devices acceptable and beneficial [27, 28]. Both patients and therapists identified challenges, including the time required to set up the system [27, 29], skin irritation, or unmet expectations [28].

Based on our preliminary searches, to date, no systematic reviews of qualitative studies exploring the end-user experiences of robotic devices in motor rehabilitation have been published. Exploring the participants’ expectations, experiences and satisfaction with the use of such devices in depth is crucial to identify potential gaps in the design and implementation of existing robotic devices and/or interventions and provide suggestions for future uptake of the technologies. This work would allow us to better understand the needs and preferences of people with motor difficulties undergoing motor rehabilitation, as well as explore any potential facilitators and barriers to their recovery. Exploring participants’ experiences might also allow us to better understand why some past studies have not shown a significant effect of robotic rehabilitation on outcomes [19] and, hence, to help identify ways to improve existing technologies and care practices.

### Theoretical perspective

We used the extended Unified Theory of Acceptance and Use of Technology (UTAUT2) [30] to facilitate our data gathering, analysis and interpretation of the experiences of people using robotic devices in motor rehabilitation.

UTAUT2 was developed to synthesise early technology acceptance research and create a model to accurately predict future consumer use of technology. According to UTAUT2, the main direct determinants of an end-user’s acceptance of and behavioural intention to use technology are: (1) performance expectancy (the degree to which the end-user believes that using the technology will be beneficial in performing certain activities); (2) effort expectancy (the degree of ease associated with use of the technology); (3) social influence (the degree to which the end-user believes that using the technology is seen as important by significant others in their life, such as family and friends); (4) facilitating conditions (the degree to which the end-user believes that enough resources and support exist to help them use the technology); (5) hedonic motivation (the degree to which the end-user believes that using the technology is fun or pleasurable); (6) price value (the degree to which the end-user believes that the benefits of the technology are worth the financial costs of using the technology); and, (7) habit (the degree to which a repeated behaviour has become automatic, mainly due to learning). In addition, individual differences (such as age, sex, and experience) are believed to moderate the relationships between the above UTAUT2 variables.

## Aim and research question

The aim of this study was to review end-users’ (patients, carers and healthcare professionals) experiences with robotic devices in motor rehabilitation, by conducting a systematic review and thematic meta-synthesis of qualitative studies in the area. Our research question was “What are patients’, their carers’, and healthcare professionals’ perceptions of and/or experiences with robotic interventions in motor rehabilitation?”.

## Methods

We followed ENTREQ guidelines for enhancing transparency in reporting the synthesis of qualitative research [31]. The review protocol was registered with the PROSPERO International prospective register of systematic reviews (PROSPERO 2019 CRD42019137852) and is available from: http://www.crd.york.ac.uk/PROSPERO/display_record.php?ID=CRD42019137852.

### Inclusion criteria

Studies were eligible for inclusion if they had a qualitative research design (e.g., interviews, focus groups, ethnography) and reported on the experiences and perceptions of patients who have undergone motor rehabilitation that involved a robotic intervention. The views of the family or carers of a patient, as well as the views of healthcare professionals involved in the delivery of the intervention (such as physiotherapists, neurologists, occupational therapists, etc.), were also of interest. Mixed methods studies that included qualitative elements were also included. Only peer reviewed studies, written in English (due to lack of resources), were considered for eligibility.

Purely quantitative studies were not eligible for inclusion, since we were interested in participants’ lived experience of motor rehabilitation that involved robotic interventions that included in depth accounts of their experiences (preferably expressed in their own words, i.e., by using quotes).

### Information sources and search strategy

Electronic database searches were performed in the following electronic bibliographic databases: MEDLINE, CINAHL, Academic Search Complete, The Cochrane Library (Cochrane Database of Systematic Reviews), PROSPERO, Scopus, IEEE Xplore, Knovel, and ACM Digital Library. All databases were searched from inception to August 2020. Searches were supplemented with internet searches (i.e., Google Scholar), as well as forward and backward citation tracking from the included studies and review articles.

The search strategy used in the above databases included a combination of two sets of keywords and related terms: (1) robotic and robot-assisted, interventions, therapy, and rehabilitation; combined with (2) qualitative research, interview, focus group, experiences, perceptions, attitudes, and views. The search terms were entered using Boolean operators and truncation. Medical Subject Headings (MeSH) were also employed in forming the search strategy. For the full search strategy used for the Medline database, see Table 1.

**Table 1:** Search strategy for MEDLINE database

Search ID	Search terms
S1	robotic* OR robot* OR robotic therap* OR robot-assisted OR robot assisted OR exoskeleton* OR assistive robotic* OR walking robotic device* OR personal care robot* OR medical robot* OR assistive OR assistive automation OR wearable robot* OR orthotic* OR orthosis OR exoskeletal* OR exo OR end-effector OR haptic* OR robot regulation*
S2	rehab* OR intervention* OR treatment* OR therap* OR program* OR strateg* OR training OR physiotherap* OR physio-therap* OR “physio therap*” OR “physical therap*”
S3	Qualitative research OR qualitative OR interview* OR focus group* OR ethno* OR phenomenolog* OR hermeneutic* OR grounded theory OR narrative analysis OR thematic analysis OR lived experience* OR life experience*
S4	(MH “Qualitative Research”) OR “Qualitative research”
S5	S3 OR S4
S6	S1 AND S2 AND S5

### Study selection and data extraction

All references were reviewed and screened by two reviewers independently. Titles and abstracts were initially screened for relevance, and final eligibility was assessed through full-text screening against the inclusion criteria, using a pre-designed study selection form. Reviewers had to fill in the selection form for each reviewed paper and indicate whether it satisfied the following inclusion criteria: qualitative study (interview, focus group, observation); robotic intervention; targeting motor skills/functions; rehabilitation only (not activities of daily living, social companions, etc.). Reviewers also had to include a reason for exclusion. Any disagreement between the reviewers over the eligibility of particular references was resolved through discussion within the review team.

A standardised, pre-piloted form was used to extract data from the included studies for assessment of study quality and evidence synthesis. Extracted information included: study details (title, authors, date); methods (aims, objectives, research questions, study design, setting, data collection methods, outcomes, data analysis, context in terms of findings and relevant theory); intervention components (description, target area), where the paper reported findings relating to an intervention; and participants (demographics, medical condition, inclusion/exclusion criteria, method of recruitment, sample selection and sample size). One reviewer extracted data and a second reviewer checked the data extractions for accuracy. Any discrepancies were resolved through discussion and missing data were requested from study authors.

### Data synthesis

Data were entered into NVivo 12 qualitative data analysis software to facilitate analysis. We used thematic synthesis to synthesise the data, following the Thomas and Harden [32] approach. Initially, three reviewers (DL, FC, JA) independently coded the “13“ sections (and “23“ sections, where new concepts were introduced) of the included papers line-by-line, according to meaning and content, using an inductive approach. Consequently, these free codes of findings were organised into ‘descriptive’ themes that encompassed the meaning of groups of the initial codes. Finally, based on the codes and ‘descriptive’ themes and through discussion with the wider review team, the final ‘analytical’ themes were developed.

### Quality assessment of studies

The Critical Appraisal Skills Programme (CASP) Qualitative Checklist [33] was used to assess the quality of the included studies of this review. The CASP qualitative checklist aims to assess various elements of qualitative research studies, including research aims, appropriate methodology, research design and strategy, methods of data collection and communication between researchers and participants, ethical considerations, rigor of data analysis, and the clarity and value of study findings.

Two reviewers independently assessed the quality of the included studies. Discrepancies were resolved by discussion and consensus among the reviewers. Low quality, however, was not a criterion for exclusion of a study since we were interested in the synthesis and interpretation of all relevant and sufficiently rich data. Instead, it was decided that any papers assessed to be of low quality would still be included in the review and relevant implications would be considered in the results and discussion sections.

### Reflexive statement

When conducting qualitative research and/or analysis it is crucial for researchers to try to make sense of the assumptions and preconceptions they bring into the research and may influence the research process and allow the reader to understand the dynamics between the researcher and the researched.

DL, a psychologist by background and a researcher in health services, has experience in quantitative and qualitative systematic reviews, as well as in the analysis of qualitative data. FC is a research fellow whose research predominantly focuses on non-pharmacological interventions for the prevention and management of chronic conditions. FC has experience conducting systematic reviews of both quantitative and qualitative studies. JA has a background in clinical nursing and public health with expertise in qualitative and quantitative research methods, and systematic reviews. KG is a robotics engineer and scientist with special focus on supporting transition from discovery research to patentable engineering innovations with high technology reediness level for assistive living. KG is experienced in users-centred design and development of assistive technologies. ANS is a clinical academic general practitioner (GP) by background with expertise in social science methods, including systematic reviews, qualitative meta-syntheses and qualitative studies more generally. AK is a computer scientist and a robotics researcher; whose focus is on machine intelligence and interaction studies. AK is experienced in quantitative data analysis.

## Results

**Fig. 1:**
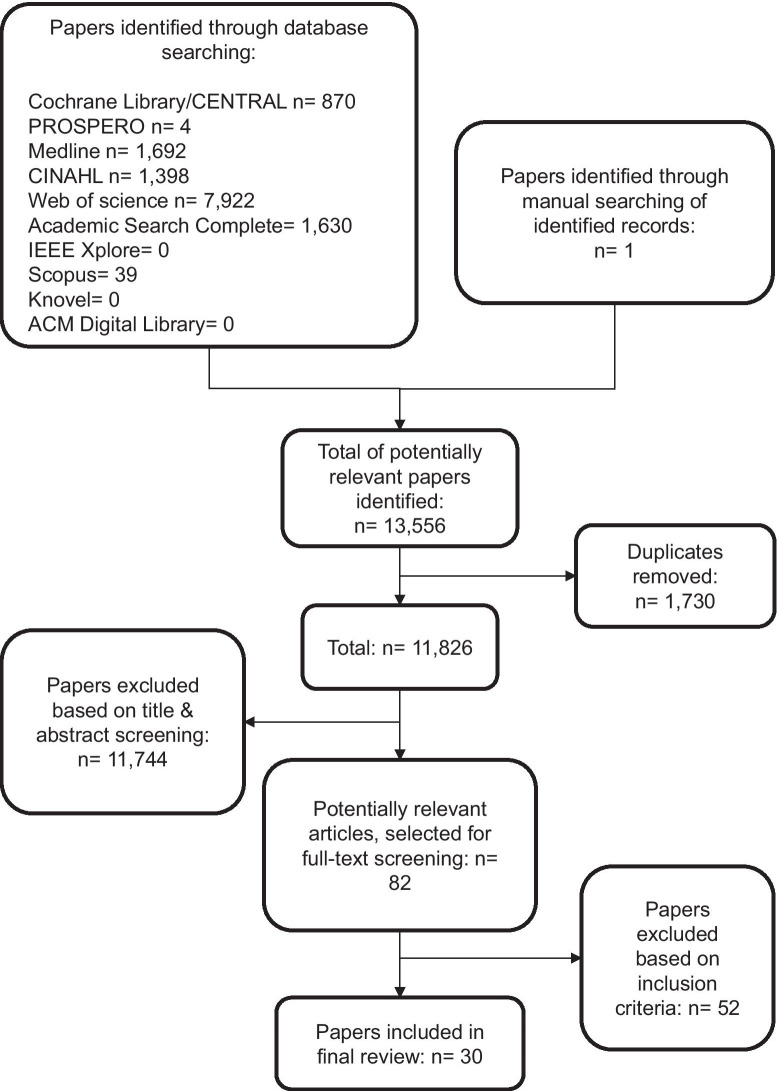
Flowchart illustrating the results of the selection process

The search strategy identified a total of 13,556 citations. After removing duplicates and excluding citations based on title and abstract, 82 articles remained for full-text screening. A further 52 articles were excluded based on inclusion/exclusion criteria, leaving 30 studies to be included in the review and meta-synthesis. Figure 1 presents a flowchart illustrating the results of the selection process.

### Characteristics of included studies

The 30 included studies (Table 2) were published between 2011 and 2020 and were mainly from eight countries: Canada [26, 34–40], USA [28, 29, 41–45], the UK [46–52], Australia [53], Turkey [54], Ireland [25], Germany [27], and the Netherlands [55]. One study [56] took place across various geographical areas (Asia, Australia, Europe and USA), one study [57] across three countries (Italy, UK, and the Netherlands), whereas another study [58] mentioned being conducted in three European Union (EU) countries, without specifying the countries.

**Table 2:** Study characteristics of reviewed studies

Study	Aim(s)	Design	Sample	Condition and target area of rehabilitation	Method of data collection and analysis
Ates et al., 2014 [58]; Three EU countries (unspecified)	To report on the technical challenges presented by the use of SPO and the feedback from therapists and patients	Technical & performance evaluation	24 patients; 33–81 years; 11 m and 9 f No information about the therapists	Stroke; hand impairment	Clinical observation and descriptive summary into themes
Beveridge et al., 2015 [34]; Canada	To explore the experiences and perspectives of parents whose young, ambulatory children with CP were undergoing Lokomat gait training, and consider how parents' values about walking influenced therapy decisions for their children	Qualitative descriptive design	5 mothers and 1 fathers of 5 (4 m and 1 f) children; children aged 5–11 years; 2 parents were Anglo-Canadian and the others from Asian backgrounds	Cerebral palsy; walking rehabilitation	Individual, semi-structured, face-to-face interviews; followed the Dierckx de Casterle approach to analysis of qualitative data: the Qualitative Analysis Guide of Leuven (QUAGOL)
Bezmez and Yardimci, 2016 [54]; Turkey	To explore the role of a robotic gait training device (Lokomat) and its role in rehabilitation in Turkey	Ethnographic study	42 participants; 7 doctors, 2 nurses, 2 physiotherapists, 2 non-medical personnel, 20 in-patients, and 9 former patients	Traumatic injury or illnesses; bodily disability and inability to walk	Individual, semi-structured interviews; no information provided on the method of analysis
Cahill et al., 2018 [25]; Ireland	To gain an understanding of the experience of using a RWD within a gym-based setting from the perspective of non-ambulatory individuals with SCI	Qualitative methodology	4 males and 1 female; mean age 40.75	Spinal cord injury; walking rehabilitation	In-depth semi-structured interviews; thematic analysis
Danzl et al., 2013 [41]; USA	To investigate the feasibility of combining tDCS to the LE motor cortex with novel locomotor training to facilitate gait in subjects with chronic stroke and low ambulatory status; and to obtain insight from participants and their carers to inform future trial design	Mixed methods: a double-blind, sham-controlled, randomized quantitative design and an exploratory descriptive qualitative design	Final sample 8 patients; 4 males and 4 females; mean age of 67.8 years (range, 44–80 years)	Stroke; lower limb (gait) rehabilitation	Semi-structured interviews; inductive thematic analysis
Eicher et al., 2019 [27]; Germany	To identify differences regarding usability, acceptability, and barriers of usage of a robot-supported gait rehabilitation system between a younger and older group of patients with gait impairments	Pilot/feasibility study assessing system usability of a robot-supported gait rehabilitation system between a younger and older group of patients with gait impairments	13 completed all sessions; 7 were older patients (group G: 75 mean age, range 69–84), 6 were younger patients (group Y: 36 mean age, range 20–55); 11 male and 2 females	Stroke/brain haemorrhage, hemiplegia, other (e.g., accidents, falls, not specified); gait rehabilitation	Structured interviews; qualitative content analysis by Mayring (2010)
Elnady et al., 2018 [35]; Canada	To describe users’ perceptions about existing wearable robotic devices for the upper extremity; identify if there is a need to develop new devices for the upper extremity and the desired features; and to explore obstacles that would influence the utilization of these new devices	Exploratory focus group discussions	Group 1: 8 people with stroke (1 f and 7 m); Group 2: 8 therapists: 4 Physiotherapists, 2 Occupational therapists; 2 Rehabilitation assistants (8 f)	Stroke; upper limb rehabilitation	Focus groups; thematic analysis
Flynn et al., 2019 [53]; Australia	To explore occupational therapists’ and physiotherapists’ perceptions of robotic therapy for the upper limb and the perceived barriers and enablers influencing implementation	Qualitative methodology involving two discipline-specific focus groups	12 participants: 6 occupational therapists (mean age = 29 years, range = 24–39) and 6 physiotherapists (mean age = 30, range = 23–51); 9 female and 3 males	Stroke; upper limb movement at the shoulder, elbow and hand (with the wrist fixed in neutral or pronation)	Focus groups; data were deductively analysed using the Theoretical Domains Framework (TDF)
Gilbert et al., 2018 [46]; UK	To determine whether or not the MUJO System was acceptable to patients with shoulder dysfunction and their rehabilitation professionals	Qualitative study	10 patients (median age was 38.5 years, range = 19–54; 5 f and 5 m) and 7 physiotherapists	Shoulder instability (n = 6) and rotator cuff related pain (n = 4); rehabilitation of the rotator cuff muscles (bi-articular muscles or multiple axial joints)	Interviews; Directed Content Analysis was undertaken to organise the qualitative data according to the four constructs of Normalisation Process Theory (NPT)
Heinemann et al., 2018 [28]; USA	To describe clinicians’ experiences, evaluations, and training strategies using exoskeletons in rehabilitation and wellness settings	Qualitative focus groups	30 healthcare professionals: 90% Physical therapist, 2% Administrative, 8% Other clinical role (exercise therapist, recreational therapist); mean age was 37, range = 28–53; 86% female	Spinal cord injuries; Standing and gait rehabilitation	Focus groups; thematic analysis
Heinemann et al., 2020 [42]; USA	To describe appraisals of robotic exoskeletons for locomotion by potential users with spinal cord injuries, their perceptions of device benefits and limitations, and recommendations for manufacturers and therapists regarding device use	Qualitative focus groups	35 patients; mean age = 48; 17% female	Spinal cord injuries; Gait rehabilitation	Focus groups; thematic analysis
Hochstenbach-Waelen and Seelen, 2012 [55]; The Netherlands	To identify criteria and conditions that people, involved in development of rehabilitation technology for upper limb training of stroke patients, should take into account to achieve a (more) successful implementation of the technology in daily clinical practice	Literature search and interviews	6 senior physiotherapists and occupational therapists	Stroke; upper limb rehabilitation	Semi-structured interviews; method of data analysis was not reported
Hughes et al., 2011 [47]; UK	To understand the stroke participants’ experiences of using the novel combination of a robotic arm and iterative learning control system and to gain greater insight into how systems might be improved in the future	Mixed-methods study, involving open-ended and closed questions	5 patients; mean age was 52 years (range = 38–77); 3 males and 2 females	Stroke; upper limb rehabilitation	Two ways data were collected: comments were recorded during the time when participants were receiving the intervention and immediately following the clinical study, an interview based question set was used; content analysis
Huq et al., 2012 [36]; Canada	To develop a portable robotic system with a haptic interface that facilitates the concept of rehabilitation at a remote location, e.g., at a home; to develop a GUI that integrates different control techniques and VR games in the same screen, and allows therapists to easily interact with the system; and to evaluate the current system with therapists in a focus group study	Focus group study	3 physiotherapists and 4 occupational therapists	Stroke; upper limb rehabilitation	Focus groups; summary of findings
Kumar and Phillips, 2013 [48]; UK	To explore the views, experiences, benefits, and difficulties that users of one specific type of PMAS perceive, and determine which areas of daily life they are used in	Mixed-methods approach, including questionnaire and interviews	13 patients; age range = 13–69 years (12 men and boys, mean age 25 years, mode age 14 years; and 1 woman, 69 years)	Neuromuscular conditions; upper limb rehabilitation	Semi-structured interviews; thematic analysis
Lajeunesse et al., 2018 [37]; Canada	To present the perspectives of individuals with ASIA C or D incomplete SCI concerning the usability of lower limb exoskeletons to R&D engineers and clinicians working in motor rehabilitation	Exploratory qualitative research design with a user-centred approach	13 patients; 6 males (mean age = 59.8) and 7 females (mean age = 43)	Incomplete spinal cord injury; lower limb rehabilitation	Individual, semi-structured interviews; inductive thematic analysis
Lo et al., 2020 [56]; Asia, Australia, Europe and USA	To inform rehabilitation clinicians about the various aspects of adopting and integrating robotic stroke therapy into clinical settings	Qualitative description design	8 rehabilitation therapists	Stroke and other neurological conditions, such as spinal cord injury, multiple sclerosis (MS), brain tumours and traumatic brain injuries; upper and lower limb training	Semi-structured interviews; qualitative descriptive analysis
Manns et al., 2019 [26]; Canada	To explore the expectations and experiences of persons with spinal cord injury, training with the ReWalk exoskeleton	Qualitative research design	11 patients (mean age = 37.5, range = 18–65 years); 4 females and 7 males	Traumatic spinal cord injury; standing and walking training	Semi-structured interviews; thematic analysis
Mortenson et al., 2020 [38]; Canada	To explore the experiences of physiotherapists with the introduction of an exoskeleton as a gait retraining device in a Canadian rehabilitation centre	Longitudinal qualitative study	10 therapists (one group of therapists, n = 4, was formally trained using the device, whereas the other group only had clinical exposure to the device, n = 6); mean age = 40 years; 8 females and 2 males	Brain and spinal cord injuries; gait training	Semi-structured interviews; thematic analysis
Nasr et al., 2015 [57]; UK, Italy and the Netherlands	To examine stroke survivors’ experiences of living with stroke and with technology in order to provide technology developers with insight into values, thoughts and feelings of the potential users of a to-be-designed robotic technology for home-based rehabilitation of the hand and wrist	Interdisciplinary research design and qualitative study	10 households (10 patients and 8 carers); age range = 60–77; 7 males and 3 females	Stroke; upper limb rehabilitation	Application of qualitative methods such as in-depth interviews as well as using diaries and photography activities; thematic analysis
O' Brien Cherry et al., 2017 [43]; USA	To determine participants’ general impressions about the benefits and barriers of using RT devices for in-home rehabilitation	Qualitative study design employing ethnographic- based anthropological methods	10 veterans (plus their carers); age range = 49–88; all males	Stroke; upper or lower limb impairments	Direct observations and semi-structured interviews; inductive thematic analysis
Phelan et al., 2015 [39]; Canada	To investigate the expectations and experiences of children with CP in relation to robotic gait training using the Lokomat Pro	An interpretivist qualitative design	5 children (8–11 years; 3 boys and 2 girls) and their parents (28–52 years; 3 mothers and 2 fathers);	Cerebral palsy; gait rehabilitation	Observations during sessions, semi-structured interviews with parents and use of a customizable “toolbox” of age-appropriate child-friendly techniques; thematic analysis
Read et al., 2020 [40]; Canada	To explore how the training and implementation of using the Ekso robotic exoskeleton with patients affects physiotherapists’ work	An exploratory qualitative research design	3 physiotherapists	Individuals with SCIs and hemiplegia due to stroke; gait training	One-on-one semi-structured interviews; thematic analysis
Sivan et al., 2016 [49]; UK	To evaluate the ICF as a framework to ensure that key aspects of user feedback are identified in the design and testing stages of development of a home-based upper limb rehabilitation system	Qualitative study	17 patients and 7 physiotherapists and occupational therapists	Stroke; upper limb rehabilitation	Face-to-face semi-structured interviews; analysis based on the updated International Classification of Functioning, Disability and Health (ICF) linking rules and core set categories
Stephenson and Stephens, 2018 [50]; UK	To explore physiotherapists' experience of using RT in rehabilitation of the upper limb, within a stroke rehabilitation centre	Phenomenological approach/qualitative study	6 physiotherapists; 3 males and 3 females	Stroke; upper limb rehabilitation	Semi-structured interviews; thematic analysis
Swank et al., 2020 [44]; USA	To describe therapists’ clinical practice experiences with robotic gait training (RGT) over 3 years during inpatient rehabilitation	Mixed methods study, including a survey and semi-structured focus group	10 physical therapists	Condition not specified; gait training	Semi-structured focus group; thematic analysis
Swank et al., 2020b [29]; USA	To determine the feasibility of integrating the Ekso Gait Training device into inpatient rehabilitation in a neurologic population	Longitudinal cohort design, including a focus group (therapists) and survey (therapists and patients)	Physical therapists (exact number not reported)	Stroke and SCI; gait training	Semi-structured focus group; thematic analysis
Sweeney et al., 2020 [51]; UK	To understand user perceptions in order to explain low uptake of upper limb rehabilitation interventions after stroke in clinical practice within the National Health Service (NHS Scotland)	Qualitative study using a mixed-methods approach, consisting of a cross-sectional online survey with therapists and semi-structured interviews with stroke patients	8 stroke patients from a Constraint Induced Movement Therapy (n = 2) and Robotic Therapy (n = 6) groups	Stroke; upper limb rehabilitation	Semi-structured interviews; thematic analysis
Tedesco Triccas et al., 2018 [52]; UK	To explore views and experiences of people with sub-acute and chronic stroke that had previously taken part in a randomised controlled trial involving tDCS and RT for their impaired upper limb	A structured and semi-structured interview study, involving qualitative and quantitative components	21 patients; 12 males and 9 females; mean age was 64.2	Stroke; upper limb rehabilitation	Interviews involving open questions; thematic analysis
Thomassen et al., 2019 [45]; USA	To generate new knowledge regarding user experiences of standing and walking with Ekso™ (Ekso Bionics, Richmond, CA, USA)	Qualitative (interview) study	3 patients; all male of different ages (from young adult to middle aged)	Spinal cord injury (due to traumatic and non-traumatic reasons); standing and walking training	In-depth interviews in a phenomenological tradition; systematic inductive content analyses

*ASIA* American Spinal Injury Association, *CP* cerebral palsy, *f* female(s), *GUI* graphical user interface, *ICF* International Classification of Functioning, Disability and Health, *LE* lower extremity, *m* male(s), *PMAS* powered mobile arm support, *R&D* research and development, *RT* robotic therapy, *RWD* robotic walking device, *SCI* spinal cord injury, *SPO* SCRIPT Passive Orthosis, *tDCS* transcranial direct current stimulation, *VR* virtual reality

Sample sizes (approximately n = 393; two studies [29, 58] did not report the number of therapists involved) ranged from three to 42 participants and most studies contained both men and women. According to the information provided, there were more male (n = 163) than female (n = 129) participants, while two studies included only male participants [43, 45]. Eight studies did not report any data on gender [29, 36, 40, 44, 49, 51, 55, 56]. Participants’ ages ranged from 8 to 88 years. Only three studies [28, 34, 42] provided information on the participants’ ethnic background, with participants identifying as White, Black, Asian, Hispanic/Latinx or multiracial. The sample consisted of patients undertaking or who had undertaken in the past robotic therapy (n = 255) [25–27, 34, 35, 37, 39, 41–43, 45–49, 51, 52, 54, 57, 58], parents (n = 11) [34, 39] or other carers (n = 8; the paper does not specify the carers’ relationships to the patients) [57], as well as healthcare professionals (n = 119), including physiotherapists or occupational therapists (n = 108) [28, 29, 35, 36, 38, 40, 44, 46, 49, 50, 53–56, 58], doctors (n = 7) [54], nurses (n = 2) [54], and non-medical personnel (n = 2) [54].

The majority of studies included patients (or their carers or their healthcare professionals) who had received rehabilitation after stroke (15 studies) [29, 35, 36, 40, 41, 43, 47, 49–53, 55, 57, 58], or brain and/or spinal cord injury (8 studies) [25, 26, 28, 29, 37, 38, 42, 45]. One study included children with cerebral palsy and their parents [39] and a second study [34] included only the parents of children with cerebral palsy. The remaining studies included conditions such as shoulder instability or rotator cuff-related pain [46], neuromuscular conditions [48], and physical disability through traumatic injury or illness [54]. Three studies [27, 44, 56] included participants with various reasons for rehabilitation, including stroke, spinal cord injury, multiple sclerosis, brain haemorrhage, hemiplegia, or any other condition (e.g., accidents, falls, not specified).

Most studies involved upper limb (10 studies) [35, 36, 47–52, 55, 57] or walking/standing/gait rehabilitation (14 studies) [25–29, 34, 38–42, 44, 45, 54]. One study [37] involved lower limb rehabilitation, one study [53] focused specifically on wrist and hand rehabilitation, one study [46] targeted rotator cuff muscles, whereas two [43, 56] focused on upper and/or lower limb rehabilitation.

Most studies were based on individual semi-structured interviews, whereas seven studies included focus groups [28, 29, 35, 36, 42, 44, 53]. One study [58] reported using clinical observations as their means of collecting data, one study [27] conducted structured interviews, one study [43] combined direct observations with semi-structured interviews, whereas another study [57] combined in-depth interviews with using diaries and photography activities. Finally, one study [39] combined direct observations with semi-structured interviews with parents, as well as interviews (using child-centred methods) and activities with children.

Various methods of analysis were employed in the studies, including thematic analysis (18 studies) [25, 26, 28, 29, 35, 37–44, 48, 50–52, 57], content analysis (four studies) [27, 45–47], qualitative descriptive analysis (one study) [56], or the Qualitative Analysis Guide of Leuven (QUAGOL) approach [34]. One paper [53] deductively analysed their data using the Theoretical Domains Framework (TDF). One paper [49] described their data analysis as initially extracting meaningful concepts (i.e., linking units) from the interview responses, based on the updated International Classification of Functioning, Disability and Health (ICF) linking rules, and then linking these concepts to precise ICF core set categories. Finally, two papers [36, 58] provided a descriptive summary of their findings into themes, while two papers [54, 55] did not report their method of data analysis.

### Quality assessment of studies

Table 3 presents the results of the critical appraisal of the 30 studies, using the CASP criteria for qualitative research. All included studies were assessed to meet an acceptable level of quality.

**Table 3:** Critical appraisal/quality assessment of studies

Study	CASP01	CASP02	CASP03	CASP04	CASP05	CASP06	CASP07	CASP08	CASP09	CASP010
Ates et al., 2014	Y	Y	CT	CT	CT	N	CT	CT	Y	Y
Beveridge et al., 2015	Y	Y	Y	Y	Y	N	Y	Y	Y	Y
Bezmez and Yardimci, 2016	Y	Y	Y	Y	Y	N	Y	CT	Y	Y
Cahill et al., 2018	Y	Y	Y	Y	Y	N	Y	Y	Y	Y
Danzl et al., 2013	Y	Y	Y	Y	Y	N	Y	Y	Y	Y
Eicher et al., 2019	Y	Y	CT	CT	CT	N	Y	CT	CT	Y
Elnady et al., 2018	Y	Y	Y	Y	Y	Y	Y	Y	Y	Y
Flynn et al., 2019	Y	Y	Y	Y	Y	N	Y	Y	Y	Y
Gilbert et al., 2018	Y	Y	Y	Y	Y	CT	Y	Y	Y	Y
Heinemann et al., 2018	Y	Y	Y	CT	Y	N	CT	Y	CT	Y
Heinemann et al., 2020	Y	Y	Y	Y	Y	N	Y	Y	Y	Y
Hochstenbach-Waelen and Seelen, 2012	Y	Y	Y	Y	Y	N	CT	CT	CT	Y
Hughes et al., 2011	Y	Y	Y	Y	Y	N	Y	Y	Y	Y
Huq et al., 2012	Y	Y	CT	Y	CT	N	CT	Y	Y	Y
Kumar and Phillips, 2013	Y	Y	Y	Y	Y	N	Y	Y	Y	Y
Lajeunesse et al., 2018	Y	Y	Y	Y	Y	N	Y	Y	Y	Y
Lo et al., 2020	Y	Y	Y	Y	Y	Y	Y	Y	Y	Y
Manns et al., 2019	Y	Y	Y	Y	Y	N	Y	Y	Y	Y
Mortenson et al., 2020	Y	Y	Y	Y	Y	CT	Y	Y	Y	Y
Nasr et al., 2015	Y	Y	Y	Y	Y	N	Y	Y	Y	Y
O' Brien Cherry et al., 2017	Y	Y	Y	Y	Y	N	CT	Y	Y	Y
Phelan et al., 2015	Y	Y	Y	Y	Y	CT	Y	Y	Y	Y
Read et al., 2020	Y	Y	Y	Y	Y	N	Y	Y	Y	Y
Sivan et al., 2016	Y	Y	Y	Y	Y	N	Y	CT	Y	Y
Stephenson and Stephens, 2018	Y	Y	CT	Y	Y	N	Y	Y	Y	Y
Swank et al., 2020	Y	Y	Y	Y	Y	CT	Y	Y	Y	Y
Swank et al., 2020b	Y	Y	Y	Y	Y	N	Y	Y	Y	Y
Sweeney et al., 2020	Y	Y	Y	Y	Y	N	Y	Y	Y	Y
Tedesco Triccas et al., 2018	Y	Y	Y	Y	Y	CT	Y	Y	Y	Y
Thomassen et al., 2019	Y	Y	Y	Y	Y	Y	Y	Y	Y	Y

*Y* yes, *N* no, *CT* can’t tell

Only 8 studies [35, 38, 39, 44–46, 52, 56] included information about the researcher’s role, potential bias and influence during the development and conduct of the study (CASP Question 6: “Has the relationship between researcher and participants been adequately considered?”). In addition, some studies performed poorly on one [43, 49, 50, 54], three [28, 36, 55] or five additional CASP questions [27, 58], since not enough relevant information was reported in the papers.

### Data synthesis

After initial coding and development of descriptive themes, we developed six analytical themes, detailing the participants’ experiences with robotic interventions, from encountering barriers and facilitators to the use and implementation of the interventions to achieving improved functioning and identifying best practices moving forward. Table 4 summarises the analytical and descriptive themes identified in this systematic review. An additional table file is included as supplementary material to present the six analytical themes and corresponding quotations from the included studies in more detail (see Additional file 1).

**Table 4:** Analytical and descriptive themes

Analytical theme	Descriptive themes	
Logistic barriers	Physical environment	Need for someone to help you with device
	Challenges of set up	Transportation and accessibility barriers
	Device positioning	Cost of device is a barrier
Technological challenges	Prior technology experience affects attitudes towards new technologies	Device is cumbersome
	Technical problems with the devices	Issues with wearing and adjusting the robotic devices
	Engineering/manufacturing challenges	Other technological challenges
Appeal and engagement	Acceptance of devices	Uncertainty and cautiousness
	Robotic devices/interventions are beneficial and fun	Motivating factors for patients to use the device or intervention
	Using novel technologies for rehabilitation	What motivated clinicians to recommend use of the device/intervention
	Appealing features of the devices/interventions	Facilitators to use of the devices/interventions
	Aspects of the devices/interventions the participants did not like	
Supportive interactions and relationships	Therapist-patient relationship is important	Support from family is beneficial
	Managing patients' expectations	
Benefits for physical, psychological, and social function(ing)	Physical benefits of using the device	Psychological issues due to the device or intervention
	Psychological benefits of using the device	Some participants had mixed experiences
	Social benefits of using the device	No improvement from using devices or intervention
Expanding and sustaining therapeutic options	Limited or absent pathways for transitioning from the medical model to that of a wellness approach following early injury rehabilitation	Important to maintain human presence
	Implementation and contextual factors	Independent use of robotic devices
	Training	Devices seen as complimentary to traditional therapy
	Time management and resources	Comparisons with treadmills and wheelchairs
	Having appropriate staff	Training goals
	Costs	Therapist training
	Accessibility and funding	Design-related suggestions
	Patient suitability and screening	Personalising the devices or intervention
	Research can be limited in terms of day-to-day relevance	Other suggestions to increase uptake and engagement with devices
	What should such devices help you achieve/activities you should be able to do with a robotic device	Devices providing feedback are useful and desirable
	Appropriate settings	Other recommendations
	Importance of tailoring devices	

#### Logistic barriers

Overall, patients found robotic therapy enjoyable, but also tiring, frustrating and difficult [52]. A recurrent theme throughout the participants’ experiences with robotic interventions were the logistic barriers that they encountered.

The participants reported challenges during the installation and set-up of the devices, where they were offered one to be used in their homes. Houses were often too small or had limited space for the device to be properly installed [26, 43, 50], there were limited electrical outlets [43], or the household lacked a ‘‘good chair’’ or ‘‘high enough table’’ to accommodate the device [43, 49, 58]. The size and weight of the device was also a deterrent for current and future use for both patients [48, 49] and therapists [36]. Patients and therapists indicated that the set-up process for the device and accompanying software and applications was time consuming [29, 34, 35, 38, 44, 46, 56] and the instructions were confusing [48, 57]. As a result, patients often felt anxiety about the devices, although this feeling decreased once the sessions progressed [39].

Device positioning and attachment/detachment also caused issues to the participants, who felt that often it was difficult- as well as time consuming- to put the device on and adjust it [27, 43, 58]. Both patients and therapists also reported that they needed help when using the devices, which they saw as a barrier [26, 28, 37, 38, 43, 45, 48, 56–58]. Therapists, in addition, felt that, before deciding to participate in similar rehabilitation interventions, patients should consider whether they have someone to help them on a regular basis (noting how taxing it can be for a carer) to avoid adverse consequences [28].

Participants also considered transportation and accessibility issues as barriers to using robotic interventions [25, 43, 46, 52]. Having to travel long distances to attend a rehabilitation session [25, 43, 46, 52] and not being able to have the devices at home was frustrating for both patients and healthcare professionals and often stopped them from using such devices [43, 46, 52]. Those living in remote areas, even if they were able to use a device at home, were affected by internet connectivity problems, causing issues with the transmission of study data to the clinical centres [43].

Finally, finances were considered a barrier as well [25, 26, 28, 35, 37, 38, 42, 50, 56]. Healthcare professionals (and patients who were able to have a device at home) were concerned about the initial cost of the device and its maintenance [28, 35, 42, 50, 56]. Patients who had to travel to access a device were concerned about travel expenses in addition to the cost of the device [25, 37, 38]. For some patients, though, cost was not considered a barrier, if buying a device allowed them to walk again [42]. One paper [46] reported healthcare professionals’ concerns that, in situations where patients can independently use the robotic devices, replacing face-to-face contact with patients would result in loss of finances for healthcare professionals and could be a further barrier to implementation.

#### Technological challenges

Patients who had prior technology experience were found to have positive attitudes towards any new technologies [57]. However, technological challenges were identified as one of the major barriers of using robotic devices and participating in robotic interventions [27–29, 34–39, 43–46, 48, 49, 51, 53, 56–58]. Participants often expressed frustration in response to the computer or software becoming unresponsive [29], acting erratically (such as the machine suddenly changing intervention levels) [43], or technical difficulties in data transmission [43]. In one study, where the rehabilitation device was accompanied by a patient app, participants found it frustrating when this app did not work well, forcing the physiotherapists to avoid using the App [46].

Technical and manufacturing issues were also identified as barriers to the use of the devices, such as the selection of the assistance levels provided by the device [49]; ergonomic issues that limit arm reach and restrict bimanual operation or fail to provide robust alignment with human limbs [48]; issues with the device battery, e.g., size and durability [37, 44]; or the whole device being (in terms of size and weight) cumbersome [27, 28, 35–37, 43, 49, 57].

The participants indicated that wearing and adjusting the robotic devices while in use was often problematic and/or uncomfortable [43, 48, 49, 58]. Specific issues were reported about the wear and tear of Velcro straps [43], the difficulty of keeping arms in place when using slings for arm supports [48], and discomfort or pain due to the strapping and positioning to secure the patient safely in the device (leaving temporary red marks or mild bruises on patients’ bodies) [27, 34, 37, 39, 56]. In some cases, the discomfort was bad enough to stop the patients from using the device [39].

Other technological challenges included patient measurement difficulties or inconsistencies [29]; problems with joystick calibration and initialisation (which required additional training by the research team), or joystick failures [49]; and issues with mechanical resistance (which is set to engage the patients to actively move their limbs) being difficult to overcome and, hence, physically straining [27]. As a result, participants felt that having technological support whenever needed was crucial for the smooth running of robotic rehabilitation [37, 38, 50, 53, 56].

#### Appeal and engagement

Patients accepted the robotic devices as ‘just another thing’ or ‘extracurricular activity’ they had to do [39], would recommend them to others and indicated a willingness to continue using these devices in their treatment [47]. Overall, participants found robotic devices and interventions useful and beneficial [26, 35, 37–40, 44, 46, 48, 49, 52, 56, 58], as well as fun and interesting [34, 39, 43, 50–52, 57]. Therapists, in addition, found robotic rehabilitation to be effective [29, 38, 56] and that it enhanced practice [40].

Participants liked the concept and purpose of the devices [49] and found the idea of using novel technologies for rehabilitation appealing [44, 49, 50, 57]. Other reasons why the devices were found attractive include: their ease of use, although many patients had no prior knowledge of and experience with similar technological devices [27, 37, 43, 57]; their external appearance, such as having no exposed cables [49]; their ability to enable higher intensity training with more repetitions [44, 49, 50, 56] and to allow patients to focus on movement without being impeded by the device’s or their own weight [45, 51, 56] or balance problems [40]; levels becoming progressively harder, boosting the patients’ interest [49]; and the feeling of external support and stability that the devices provided [27, 52].

Physiotherapists appreciated how robotic devices enabled them to treat patients, such as those who have had strokes with contraversive pushing, when more traditional approaches would be challenging, if at all possible [44]. Therapists commended that the devices can help patients achieve more repetitions [49], allow targeted rehabilitation (e.g., by breaking down the gait pattern) [38], and provide accurate data to determine training needs and assess the patient progress [56]. They felt that having adjustable assistance/resistance levels, based on individual performance or deficits, was especially beneficial for patients [44, 49]. Therapists also felt that one of the main benefits was the reduction in physical exertion and strain due to the devices doing most of the required (hard) work [38, 40, 44, 56].

Nonetheless, there were cases where participants had mixed impressions; for example, finding devices fun, boring and uncomfortable/painful at the same time [39]. Participants also indicated aspects of the robotic devices and/or interventions that they disliked [26–28, 34, 37–40, 42, 45, 46, 48–52, 56, 57]. Patients, for example, commented that the devices looked “strange” and like a robot or a “transformer” [27, 37, 48], whereas they would prefer it to be camouflaged and look more human-like [37]. Participants did not like when the machine made them walk too slowly, “like a robot” [37]; complained that robotic therapy became boring quickly [26, 34, 39, 49, 50]; or that it was not challenging enough [39]. Others did not like that the intervention was too exhausting [26, 56]; that they could not control or feel almost forced into standing and unable to reverse or stop, even when feeling pain or a sudden increase in spasticity [45]; that the device felt unnatural to wear [45] and that walking without the device made them feel more free [39]. Some participants, who interacted with games during the therapy, found the games frustrating, not fun or not challenging enough [51, 56]. Some patients did not like the computer graphics of the video games, which they felt were ‘not accurate, nor well designed resulting in feelings of confusion’ [52]; whereas others disliked the unappealing format of the feedback, such as bars and charts, and would prefer to receive feedback in the form of simple scores [57].

Some participants (both therapists and patients) were sceptical about the value of exoskeletons due to the current state of robotic technology in general not being sufficiently advanced [42] or due to specific constraints of a device (e.g., limited terrain that the device could be operated on) [42] and felt that devices were still in the early days of development [38, 42]. Therapists, in particular, were cautious, emphasizing the need to first see the effectiveness of the device for themselves before committing to using it with patients [53], and were unsure about how robotic rehabilitation would fit within current practice, given the overall complexity of the technology [38]. Some therapists also thought the exoskeletons felt disembodied and robotic [38]. Physiotherapists discussed the time constraints of having a patient use a robotic device within a typical physiotherapy session (often due to lengthy set up procedures) and showed preference to other means of exercise (e.g., using a treadmill), which would allow the patients to spend more time rehabilitating [28, 46].

Despite these objections, participants felt motivated to use the robotic devices [26, 28, 34, 37, 41, 46, 49–54, 56, 57]. For patients, the main motivation was getting better and improving their functions [26, 28, 34, 37, 41, 50, 54, 56, 57]. Other motivators were attending sessions, as patients felt they could not motivate themselves to the same extent at home and needed the extra ‘push’ [51]; receiving performance feedback [49, 50, 52, 53, 56, 57]; involving others and having human interaction while using the device [57]; competing with others, but also with themselves and their previous performances [51, 57]; as well as participating in an intervention that they viewed as an innovative means of facilitating a return of function (instead of a pill, for example) [41], and participating in a research study in general [49]. For therapists, the main motivation for suggesting the use of robotic devices/interventions was their belief that having their patients participate in such task would release clinician time and promote patient compliance, by motivating patients to work on other functional tasks that they might be less inclined to do [28, 46].

Participants also discussed factors that facilitated their use of the devices or their participation to a robotic intervention. For example, patients felt that they needed to be physically fit to use and bear the devices, and, hence, considered the timing important for starting to use the robotic devices, in relation to their disease severity and physical condition [48, 52]. Patients mentioned that being able to access the machine on a regular basis, and perhaps for a longer period, would optimise their outcome [46, 49].

#### Supportive interactions and relationships

One thing that further facilitated device use and improved the participants’ experience was having supportive relationships in their lives. Therapeutic relationships, in particular, appeared to form the foundation for successful participation in robotic rehabilitation and person-centred intervention [25, 28, 34, 39, 50, 52, 57].

Healthcare professionals’ positive attitude was generally seen as a crucial factor in enabling progress with therapy and especially in motivating the patients to use the devices [25], while positive interactions with the healthcare professionals facilitated the success of the sessions [39, 52]. In addition, having regular [57] and enjoyable [34] interactions with a caring, supporting, and reassuring professional [28, 39] was viewed as essential for a successful recovery.

Healthcare professionals mentioned that patients often have unrealistic expectations about how much their abilities can improve after motor rehabilitation and about what robotic devices can do [28, 38, 40, 48, 54]. Therefore, clinicians emphasized the importance of open and honest conversation with patients about both their prognosis and the capabilities of the robotic device [28, 38, 54]. However, they also highlighted the need to encourage the patient’s motivation and positive attitude, as being categorical about the level of improvement can also have adverse effects and lead to patients abandoning all efforts, becoming depressed and/or aggressive [54].

Having supportive family relationships was also beneficial to patients, for both children and adults [34, 39, 54, 57]. Involving a family member or friend in the rehabilitation training was observed to increase motivation and engagement [57]. Especially when the participants were children, parents often participated in rehabilitation as stand-by coaches and motivated their children to continue their efforts. Parents valued walking and felt that walking ability, proficiency and quality was vital for their child’s wellbeing [34]. When commended by their parents, the children were observed to be happy and proud, and so were the parents in their roles as motivators [39].

#### Benefits for physical, psychological, and social function(ing)

Both patients and healthcare professionals described how using a robotic device helped patients to improve functional ability to perform basic ADLs (such as eating or dressing independently, grasping items, etc.) and their physical functioning [25–28, 37, 39–41, 43, 47–52, 56, 57]. Using a robotic device helped patients improve movement [43, 49, 51, 56, 57] and/or gait/walking/standing [26–28, 39–43, 45, 47, 56], regain muscle strength [44, 47, 49, 51, 57], and improve balance and fitness [25]. Participants also felt that robotic devices helped prevent falls [27, 34] and promoted independence [25, 34, 43, 49, 57]. Other (indirect) health benefits from using robotic devices were improvements in spasticity, cardiorespiratory function, circulation and sensory feelings, pain, bowel and bladder function, urinary tract infections, [25, 26, 28, 29, 45], as well as wound healing [25]. In one study [46], physiotherapists indicated that the robotic device can also be used successfully as an assessment tool for the patient’s condition and progress before and after the intervention.

Psychological benefits of using the robotic devices were also reported [25–28, 34, 39–43, 45, 48, 49, 52, 56]. These included self-reported mood improvements by the patients [25, 43, 49], a sense of achievement [25, 28] and empowerment [44, 45], as well as increased confidence and self-esteem [26, 39, 41, 48]. Being able again to do everyday things, like before or like others, brought joy to patients and made them realise how much they had missed things like being able to stand on the same level as others or go for a walk [26, 45, 56]. Using the robotic devices also returned the hope for a full recovery to participants [26, 28, 38, 49, 53] and gave them a sense of purpose in life and something to look forward to each day [26, 43, 49, 52]. Other psychological benefits reported by patients were an improved body-mind connection (i.e., an increased awareness of and better sense of connection with the affected area) [25, 43]; improved sleep quality [25]; as well as reduced mental tension, anxiety, and frustration [43].

In addition, patients reported social benefits of using robotic devices and taking part in a robotic intervention, such as the opportunity to converse with other participants, as well as increased energy and mobility to interact with others in general [25, 26]. Therapists also reported that patients felt more engaged in social situations, due to being able to stand and maintain eye contact or hear better when in a conversation [28].

Nonetheless, not all participants had a completely positive experience with using robotic devices. For example, some participants reported feeling anxious, fearful and insecure about using a robotic device, especially in the beginning of their treatment; these feelings were often attributed to fear of falling, uncertainty about what could happen while attached to the robotic device or having to give up some motor control to the device [26, 37, 39, 40, 44]. Patients felt embarrassed or self-conscious when using one in public [37, 48], although often such negative feelings were outweighed by the perceived benefits of using the device [48]. Participants also reported that using a robotic device made them feel weak and was a constant reminder that they still had to deal with their impairment [25, 26, 35, 45, 48, 52]. Some patients found challenging the temporality of positive sensations after training [45] and some even felt sad and disappointed at the end of training, feeling akin to “getting fired” [26]. Others worried about the long-term implications of using an assistive device, as they believed that relying on such a device would compromise their independence; as a result, some patients refused to use assistive devices [35]. In one study, therapists noted that they did not have a sense of patient ownership and felt that they had become technicians and were no longer clinicians, mainly due to the way robotic training was organized [56]. Finally, some therapists complained about additional cognitive or mental workload, due to the training and subsequent therapy sessions of robotic rehabilitation [38, 40].

Furthermore, some participants (including patients, carers and physiotherapists) reported having mixed experiences with the devices and were ambivalent about its benefits [34, 39, 50, 52]. Participants, for example, had difficulty making an explicit functional link between using the device and an improvement in function [34, 39, 50]. In some cases, this perceived lack of improvement was also regarded to be a result of the patients’ severe impairments, making it harder for them to regain as much movement as they would have liked [52]. Interestingly, though, other participants (both patients and clinicians) felt that perhaps the robotic devices would be more suitable and effective precisely for people with more severe impairments [27, 49]. Finally, there were some cases where participants did not notice any improvement in their functions, but no justification was given for the lack of improvement by the authors of the papers [49, 51].

#### Expanding and sustaining therapeutic options

Participants also made recommendations for future use and development. Patients, for example, expressed a need for transition from a medical to a wellness model following early injury rehabilitation, which is currently lacking [25]. Accordingly, patients were frustrated to continue being treated like a patient in the community (i.e., after initial rehabilitation following their injury) and would prefer to fully integrate into ‘normal society’ (e.g., exercising in the community in an integrated gym) [25].Moving forward, therapists stressed the importance of better pre-implementation and implementation planning and processes to ensure that appropriate and well-trained staff and systems are in place for future robotic rehabilitation interventions [53, 56]. Organisational culture (especially support from line managers) and the work environment (including staffing, technical support, location and space considerations) were believed to positively influence the implementation process and health professionals’ behaviour when implementing a new practice, such as robotic rehabilitation [53, 56].

Physiotherapists believed that their own training and education is important for the proper integration of exoskeletons into rehabilitation therapy services [28]. Therapists felt that they should receive more in-depth information about the training process and the technology, including the opportunity to practise as much as possible [38]. Many physiotherapists were worried about time constraints and the need for prioritization of workloads, in order to be able to fully engage in robotic rehabilitation [38, 40, 46, 50, 53]. They also felt that having different grades of staff would facilitate such constraints and could help with the “delivery of safe, effective and also efficient rehabilitation” [50, 56]. Others, however, felt that fully qualified staff should remain throughout a session, as a minimum, to provide guidance to less qualified staff [56]. Although physiotherapists felt that robotic devices can be expensive to produce, purchase and run [37, 38, 50], they also reported that robotic therapies may be cost-effective, especially if one physiotherapist can supervise more than one patient at a time [50]. One key point that both clinicians and patients agreed on was that there are often accessibility (including finding an appropriate setting close to home or therapists with relevant training) and funding issues from health and social care services, prohibiting people from accessing robotic rehabilitation [28, 35, 37, 46, 48, 50, 54]. Patients also felt that it “would have been more beneficial had they received the intervention earlier in their recovery process” [49, 52].

Clinicians emphasized the need for thorough screening of candidates to avoid adverse consequences, such as injury from falls [28], and suggested coordinating therapy sessions according to patients’ medical needs, such as physician-prescribed medication or incontinence [44]. Many discussions revolved around patient suitability for robotic rehabilitation. Accordingly, therapists felt that in order to be able to use robotic devices, patients would need to be in adequate physical shape (including having good eyesight) [38], have sufficient comprehension abilities [44], and be willing to participate and not be “fighting” therapy [56]. Although many participants reported that robotic devices are suitable for certain individuals only [40, 42, 53, 56], there was disagreement on how suitability should be defined. Some therapists felt that robotic rehabilitation is preferable for patients with severe impairments only [40, 56] and others suggested that newly injured patients would be more suitable [40]. In addition, some patients felt that robotic devices for gait rehabilitation would be a useful tool only for individuals who have some ability to walk [42]. As a result, patients were often disappointed that their condition/physical characteristics prohibited them from having robotic rehabilitation [42]. In addition, suitability decisions and accessing robotic training services in a clinic were often influenced by funding and the financial possibilities of each patient [56].

Physiotherapists recognised that sometimes research can be limited in terms of day-to-day relevance, especially replicating the intensity of trials in normal practice and often using outcome measures that are not sensitive enough to detect change [50]. Similarly, patients felt that sometimes their whole rehabilitation had been misleading and that what they had learned in the hospital did not prepare them for life in the world outside [54].

Patients and therapists, however, had somewhat diverging views on what their needs were; patients would prefer to have assistive devices to help them with daily life activities, whereas therapists would prefer therapeutic devices to complement traditional therapy or for use in therapy [35]. Participants went on to discuss body areas and functions that they would like to be able to train with a robotic device: hand, wrist and fingers at the same time [47, 49, 52]; hand opening and closing while performing reaching movements [49]; both distal and proximal control [35, 56]; reaching, grasping and holding objects [56, 57]; standing, stepping, and gait training [26, 28, 34, 37]; maintaining balance and walking safely [26, 34, 37]; as well as walking longer distances and improving gait speed [26, 34, 37]. Overall, participants expressed as a main goal to improve performance in ADLs, such as drinking, getting dressed, etc. [35]; doing household chores [35, 37]; going out for grocery shopping and walking the dog [37]. Patients also reported a desire to resume their social activities and hobbies, such as going to crowded places, playing with their dog, walking together with their partner, hunting, swimming, etc. [37].

Regarding the most appropriate setting for robotic rehabilitation to take place, participants had divergent views. Some participants felt that exercising in the community in an integrated gym, with physically fit people, would be preferable [25, 46, 49], whereas others would prefer to do their rehabilitation in stroke centres or together with people with similar disabilities [49]. Other participants preferred having a device for home use [43, 49, 52, 57], mainly due to convenience, even expressing a willingness to buy a robotic device they can use in their homes [49]. Finally, some patients stated that they would prefer to start their rehabilitation in a clinical setting and then, after discharge, continue use in a gym environment [25].

Participants felt that it is crucial for new devices to be tailored to each individual, as it is difficult to find a single design that would fit and work for everyone [35, 58], as well as to maintain human presence and interactions (i.e., having a therapist as well) when using a robotic device [25, 36, 39, 43, 50, 57]. Therapeutic relationships were considered the foundation for successful person-centred rehabilitation and a key ingredient for maintaining the participant’s interest and motivation, and, achieving a successful outcome (i.e., recovery). However, there were participants who preferred having therapy with just a device, as they felt that they could focus better on their exercises, avoid extra frustrations from social interactions with a therapist or their caregiver helping them [43], as well as feeling more independent [50, 57]. Finally, there were also those who felt that a robotic device should be a complimentary addition to traditional therapy sessions with a therapist [29, 35, 38, 40, 49, 50, 53, 55, 56], as well as some participants who would prefer to train with a treadmill [29] or use a wheelchair in everyday life [45].

Regarding the training itself, therapists argued that it should be patient- and goal-tailored, take into consideration the patient’s cognitive impairments, and should resemble the real-life context of patients as close as possible [55]. Training should also be motivating and challenging in order to be beneficial [25, 29, 55], and should aim to increase the intensity and frequency of meaningful task-related movements [55]. Although some therapists were satisfied with their training [38, 44], others found it too technical [38] or even inadequate [56].

Furthermore, participants made suggestions in relation to the design and ergonomics of the devices. Accordingly, it was proposed that: the controls of the devices should always be accessible, and not, for example, on your back where you cannot reach them [37]; the devices should be ‘ready to go’ and not need long set-up procedures [35, 55], as well as being lightweight and portable [26, 35, 37]; the device should have an emergency battery or a low level battery warning [37]; and in case of screen based interventions using games, end-users should be able to connect the system to their own TV and play the games, to avoid the need for an extra device in the house [57]. Other suggestions were to provide exchangeable lower leg shells [27]; facilitating movements beyond a single plane [49]; and having simple control and feedback mechanisms such as biosignals or visual cues [35].

Participants also made suggestions to increase patient engagement and uptake of the interventions, such as making games more functional, interesting, challenging, and fun [49, 56, 57]. Personalising the devices based on individual preferences and abilities was a point raised throughout the studies [27, 28, 36, 37, 48–50, 52, 55–58]. Participants suggested that patients should be given options to select levels based on their abilities, as well as set their preferences and choose games based on their individual interests, such as sports, puzzles, music, etc. [36, 49, 52, 57]. Participants felt that they would prefer having a system that can adapt to each person’s physical properties [27, 37, 49, 58], physical abilities [27, 28, 48, 49, 55, 57], and technological skills [57].

Participants suggested that: more training sessions with the devices were needed [27]; the system should give clear instructions to patients about the exercise or task to be performed in a variety of modalities, for example, both verbal and written [49, 55, 57]; and, that video or audio communication with the therapists were preferred over textual communication, as some patients may find the latter physically harder [57]. Both patients and therapists felt that receiving feedback on the patient’s performance would be beneficial [39, 49, 50, 55, 57], but only if it was given during or right after the session [55, 57], in a clearer and easier to interpret [52], and preferably culturally responsive (i.e., taking into account the users’ lifestyles, values and thoughts) [57], format. Physiotherapists felt that biofeedback in particular would be desirable, as it would allow them to have information relating to joint position, muscle use and activation [36]. Patients in one study suggested that more female patients should be included in research studies, as they had a feeling that current studies focused on men [42]. Finally, therapists stressed the importance of continuing to perform clinical work to avoid losing conventional therapy skills and to upskill as well [56].

## Discussion

This systematic review and meta-synthesis explored end-users’ experiences with robotic devices in motor rehabilitation. The findings of the review have shown that, although participants may have struggled with logistic and technological challenges initially, they quickly overcame these challenges and found the robotic devices beneficial and appealing. These results are closely aligned with concepts suggested by the extended unified theory of acceptance and use of technology (UTAUT2) [30].

Participants found robotic devices acceptable and viewed them as useful and beneficial (physically, psychologically, and socially), which motivated them to use these robotic devices further (*performance expectancy*). This was true for both patients and healthcare professionals; results which echo those found in previous studies [20–24]. Even in cases where patients had negative feelings stemming from device use (such as feeling embarrassed or self-conscious when using one in public), these negative feelings were often outweighed by the perceived benefits of using the device. On the contrary, patients refused using the devices when they felt that the disadvantages of using a robotic device outweighed the benefits (e.g., believing that relying on such a device would compromise their independence).

*Effort expectancy* also affected current and future use of robotic devices, with technological and logistic challenges deterring participants from using or recommending the devices. Time requirements of having a patient use a robotic device within a typical physiotherapy session, made physiotherapists show preference to other means of exercise, which are less time consuming. On the contrary, finding the devices easy to use made them seem more appealing and participants more willing to use them. Similar results have been found in past studies, with participants reporting frustration that robotic therapy can be time-consuming [23, 24]; that they felt tense, pressured, or nervous [24]; that the computer program and robotic device malfunctioned [23]; and that they had trouble interpreting the data on user performance [22, 23].

*Social influence* was another variable affecting the use of robotic devices. Having supportive relatives or friends and involving them in the rehabilitation training increased motivation and engagement. Especially for children, knowing how important their physical improvement to their parents was, as well as receiving encouragement from them, was a huge motivator to continue their efforts with robotic rehabilitation.

In addition, participants identified various *facilitating conditions* to their use of robotic devices, such as being able to access them earlier in their recovery, on a regular basis and for a longer period of time; having strong and positive therapeutic relationships with suitably qualified and knowledgeable therapists; having devices tailored to each patient, as well as patient- and goal-tailored training; personalising the devices based on individual preferences and abilities; having clearer instructions and the possibility of having communication with the therapists through an audio or video message; and, finally, receiving timely feedback on the patient’s performance, and perhaps biofeedback as well for therapists to have information relating to joint position, muscle use and activation. On the contrary, when participants felt that resources were lacking (e.g., time-constraints and workload for therapists), or that they didn’t have enough support to use the devices (e.g., funding or accessibility issues), they saw these as barriers to use. These results are aligned with past quantitative studies, which have also shown that end-users would like to have robotic devices that can be personalised and adapted to each user [20, 23].

*Hedonic motivation* also played an important role in participants’ level of engagement with the robotic devices. End-users found robotic therapy enjoyable, fun, and interesting, which motivated them to continue their efforts. In addition, having enjoyable interactions with therapists was considered essential for a successful recovery. In contrast, the participants’ engagement with the devices and their therapy dropped when they felt that the device is boring or unappealing. *Hedonic motivation* was particularly salient to the population interacting with games during the therapy, who recommended making games more interesting, challenging, and fun. Past studies have found similar results, with participants often finding robotic therapy enjoyable [22–24]. In one study [22], when participants reported not enjoying the therapy, they were also found to have fluctuating attention and concentration, and required cueing to remain on task, which shows the importance of keeping the participants’ interest going.

Another variable that determined the participants’ acceptance and use of the devices was *price value*. Despite considering finances a challenge, participants also felt that buying or going to therapy with a robotic device was worth it, as they felt the benefits outweighed any cost; physiotherapists found robotic devices cost-effective (especially if one physiotherapist could supervise more than one patient at a time) and there were patients who would even be willing to buy a robotic device for home use. A previous quantitative systematic review, looking into the economic cost of robotic therapy [18], showed that robotic therapy can be cost-effective, depending on the number of patients who can be treated per robotic session and the time therapists spent with patients during each session; a finding which echoes the therapists’ views from this review.

Finally, in some cases participants’ prior experience with or knowledge of technology (*habit*) affected positively their attitudes towards new technologies (i.e., the robotic devices), but in other cases lack of previous experiences did not affect their views, as participants still found the devices appealing and easy to use. Perhaps in this specific population of people undergoing motor rehabilitation and using robotic devices, the more salient feature is the novelty of the devices, which also increased their *hedonic motivation* to use the devices further and continue their efforts with their rehabilitation.

As this was a meta-synthesis of qualitative studies, it was not possible to conduct analysis of the relationships between the above variables of UTAUT2 and the participants’ individual differences (such as age, sex, and experience).

### Strengths and limitations

This systematic review has brought together papers discussing motor rehabilitation with different robotic devices, for different types of motor difficulties, as well as different end-users, and has synthesised them for the first time into a comprehensive overview of end-users’ experiences with robotic rehabilitation.

The study followed a rigorous pre-specified protocol (registered with PROSPERO), which ensured that the review process was transparent and replicable. We conducted a comprehensive search for published and unpublished work, through nine electronic databases, internet searches and scanning of bibliographies. We identified 30 studies for inclusion, sharing views and experiences from a broad spectrum of people with motor difficulties, undergoing (or being involved in) robotic rehabilitation. The quality assessment of the studies revealed that all were of acceptable quality. The final development of themes was undertaken through discussion with the wider review team, consisting of reviewers from different backgrounds (e.g., computing, engineering, medicine, nursing, and psychology), and various direct quotations from the reviewed studies were presented to enable critical appraisal of our analysis and to show how each study contributed to each theme.

The inclusion and analysis of 30 studies led to six themes and 58 descriptive themes, among which we could only select a few descriptive themes for presentation in this meta-synthesis. Future systematic reviews could aim to synthesise papers based on the neurological deficit of its users, or the type of participant (e.g., patients or physiotherapists, etc.), or even specific types of robotic training (e.g., gait or lower limb only).

### Implications for policy and practice

Synthesising this literature has allowed us to explore the acceptability of robotic devices in motor rehabilitation by different end-users and to identify possible facilitators and barriers to the use of such devices in therapy.

One of the major barriers was the various challenges encountered by end-users, especially during installation, fitting and set-up of the devices. Manufacturers should aim to reduce the time needed and facilitate the process for end-users to install, fit and set-up a device and perhaps consider having devices pre-set and “ready to go”. Systems that can be easily integrated in users’ homes, and those that can be connected to the existing home devices, such as televisions or game consoles, would have better chance for future uptake. To overcome the technological problems encountered while using the devices, appropriate and self-explanatory troubleshooting instructions, as well as continuous and long-term technical customer support, are considered an essential requirement. In general, participants were not happy with the size and weight of the devices, making them inconvenient for home use, and often needed help from others to set up and fit the devices. Hence, manufacturers should pay attention to making the robotic devices as light and as easy to put on as possible. Based on the end-users’ suggestions, batteries should also be lighter, and the straps used to secure the user to the robotic device should be more ergonomic, as it was not uncommon for patients getting physically bruised and hurt while wearing the device.

Participants from the reviewed studies also reported encountering problems of accessibility and costs, which hindered participation in robotic therapies. Given the proven, as well as perceived, benefits of robotic rehabilitation, health and social care services should consider including robotic rehabilitation in the type of therapies they provide to people living with motor difficulties, who could benefit from this technology. Based on the participants’ views, this type of therapy should be offered early in their recovery and with the possibility of accessing it regularly. Nonetheless, human interactions were considered extremely valuable by the patients; therefore, having a trained professional supervising robotic therapy would be advantageous. In addition, these technologies can be designed to get patients to interact with each other, promoting social interaction and potentially achieving better therapeutic goals [59].

Having the capability to consistently measure patient performance during repetitive and intense therapeutic interactions, robotic devices are well-suited to be integrated in rehabilitative interventions to provide objective and quantitative evaluations. In line with this, participants felt that receiving feedback on the patient’s performance would be beneficial, but only if it was given during or right after the session, if it was clearer and easier to interpret and, preferably, culturally responsive (i.e., taking into account the users’ lives, values and thoughts). Manufacturers should take into consideration these suggestions when designing relevant systems and pay attention to the visualisation and understandability of the collected data by the end-users. The general education of the health personnel in visualisation literacy is also critical to implementing robotic interventions and accompanying feedback mechanisms.

Our findings also showed that it is very important for participants to have devices that are tailored to their individual physical and cognitive capabilities, needs and abilities, and their experience with technology. Assist-as-needed control mechanisms [60–62] have been widely investigated in robotic rehabilitation research. The idea focuses on responding to variability of human neuromuscular control and uses adaptive control mechanisms rather than fixed kinematic control. The implementation of adaptive control for rehabilitative motions is not a straightforward problem, hence this stands as an open-ended research area. In addition, the estimation of human skills is a challenging research problem. Although modelling of user behaviour has been investigated to personalise interactions in different domains, such as gaming [63], assistive technologies for daily activities [64], and assisted mobility [65], their application to rehabilitation is limited. Given the need for adaptive, intelligent, and personalised robot control strategies, more research in this domain is needed. In addition, it could be mutually beneficial for manufacturers and academia to collaborate when designing devices equipped with intelligent software that allow the intervention to adapt appropriately to the end-user’s abilities, in order to increase user comfort and achieve the best outcomes for their recovery.

With the advance of virtual reality, the integration of games in rehabilitation therapy has gained momentum [66]. Participants were keen to interact with robotic devices, where they can personalise the interaction based on individual interests and preferences, such as having relevant games, involving sports, puzzles, music, etc. Games also have the potential to provide a unique motivational setting for functional improvements, where game difficulty can be mapped directly to therapy intensity. Apps and games designers and developers should collaborate to build better therapies that consider good design principles and implement dynamic difficulty adjustment where appropriate [67], in order to make robotic rehabilitation more exciting, challenging, and fun, and, hence, motivate end-users and increase compliance to robotic therapy.

Finally, it is important to understand end-user perspectives before developing therapeutic devices. Ideally, manufacturers should follow a co-creation process and conduct interview studies with their target population, so they can explore their needs and expectations, as well as the features that would be appealing to end-users, in order to better engage them and increase compliance with robotic therapy.

## Conclusions

Despite experienced technological and logistic challenges, participants found robotic devices acceptable, useful and beneficial (physically, psychologically, and socially), as well as fun and interesting. Having supportive relationships with significant others and positive therapeutic relationships with healthcare staff were considered the foundation for successful rehabilitation and recovery. Participants also made recommendations for future use and development of robotic devices and interventions, which should be taken into consideration in order to better involve end-users in the development process of robotic devices in order to increase acceptance and promote their health conditions.

## Supplementary Information

**Additional file 1:** **Table S5**. Themes and supporting quotations. A table containing the analytical themes, descriptive themes, and supporting quotations extracted from the included studies in this systematic review.

## Data Availability

This study is supported by data generated in multiple publications, which are available at locations cited in the reference section. The data supporting the findings of this study are available within the article and its Additional files.

## Abbreviations

ADLs: Activities of daily living
CASP: Critical Appraisal Skills Programme Qualitative Checklist
EU: European Union
GP: General practitioner
ICF: International Classification of Functioning, Disability and Health
MeSH: Medical Subject Headings
QUAGOL: Qualitative Analysis Guide of Leuven
TDF: Theoretical Domains Framework
UTAUT2: Unified Theory of Acceptance and Use of Technology
