# Supplementary material for: Patient, carer, and staff perceptions of robotics in motor rehabilitation: a systematic review and qualitative meta-synthesis
Source: J Neuroeng Rehabil. 2021 Dec 25;18:181. doi: 10.1186/s12984-021-00976-3 (PMC8710022; doi:10.1186/s12984-021-00976-3)
Supplement: Supplementary file 1 — Additional file 1: Table S5. Themes and supporting quotations. A table containing the analytical themes, descriptive themes, and supporting quotations extracted from the included studies in this systematic review. [file 12984_2021_976_MOESM1_ESM.docx]

**Table 5.** Themes and supporting quotations

| **Analytical theme** | **Descriptive themes** | **Supporting quotations** |
| --- | --- | --- |
| Logistic barriers | Physical environment | “Some of the problems we had in setting them up were to do with space; that was a big one” Therapist (Stephenson & Stephens, 2018) [50];  ‘‘It’s cumbersome to find the right chair because of the height and to get the monitor at the right height and close enough’’ (O' Brien Cherry et al., 2017) [43];  ‘‘Our son made an arm rest to go on the chair to make it higher than normal and wider. Before I had to hold it up and it tires the arm out fast’’ Patient (O' Brien Cherry et al., 2017) [43];  “The device took up significant space in their kitchen and we  would not wish to have such a device at home in the future.” Carer (Sivan et al., 2016) [49];  “Most therapists felt that the device was too heavy and large for home use. They found that the device is unsafe for clients to use alone, because of visible electrical wiring, but predominantly because patients may not have sufﬁcient trunk support. The device would need to have more rounded edges to prevent injury. They also suggested a longer forearm support end-effector for user safety.” Paper author/researcher (Huq et al., 2012) [36] |
|  | Challenges of set up | “Parents stated that the set-up process was often overly time-consuming” Paper author/researcher (Beveridge et al., 2015) [34];  “Essentially what’s happening is you’re spending your whole 45-minute session setting up Ekso” Therapist (Swank et al., 2020) [44];  “They managed to start the system with some prompts from the researcher but they found the instructions confusing and suggested that some texts should be reworded” Paper author/researcher (Nasr et al., 2015) [57];  “Another therapist acknowledged that using new devices is a learning process: [….] I know it takes some learning and it gets a little bit better but initially they [users] seem to perceptually have a really hard time with what exactly is going on in the exoskeleton.” Therapist (Elnady et al., 2018) [35];  “I come and I see this machine, and the set up, everything, you know, it was a bit anxiety provoking let’s say, and [Claire] didn’t feel great about it, so then I felt more anxious about it, so . . .We got off to a rocky start [Laughs] with the Lokomat […] if the machine is not cooperating, then there’s tension and everything could fall apart. [Claire’s Mother]” Patient & Parent (Phelan et al., 2015) [39] |
| Logistic barriers (continued) | Device positioning | ‘‘The hardest thing was getting my foot in it. Even with a helper it’s hard to get it in there and get my foot to fit in there right’’ Patient (O' Brien Cherry et al., 2017) [43];  “The attach and detach of the system was also criticized as being time-consuming” Paper author/researcher (Eicher et al., 2019) [27] |
|  | Need for someone to help you with device | “[. . . ] someone always has to be with you. You cannot put it on alone” Patient (Lajeunesse et al., 2018) [37];  ‘‘The only difficulty was putting it on ‘cause you needed two hands’’ Patient (O' Brien Cherry et al., 2017) [43];  “. . . screening for personal use aspect, I think what it really boils down to is us just being able to have open and honest conversation […] taking into consideration . . . is there a support person. Their 65-year old mom who might be in good shape but . . . cannot necessarily offer maximum assistance if this person needs a signiﬁcant amount of assistance . . . we have to have this conversation from the very beginning” Clinician (Heinemann et al., 2018) [28];  “One participant (participant 7) had recently stopped using his arm supports in school. When questioned further about this he recalled, ‘I didn’t want to bother at school’ since they were ‘a bit hard [for the support workers] to put them on’” Patient (Kumar & Phillips, 2013) [48] |
| Logistic barriers (continued) | Transportation & accessibility barriers | ‘‘’We’d have to go to Decatur [Georgia] from here and that’s a good 4 hours with travel time. Normally when we have a doctor’s appointment, we’d leave at around 4 in the morning so that we can get down there’. Another patient explained, ‘I wouldn’t have done the therapy if I had to go down there’’’ Patients (O' Brien Cherry et al., 2017) [43];  “The usefulness of prioritising the use of the MUJO System when it was not available locally was questioned by all clinicians: ‘It’s the repeatability. It’s them being able to learn an exercise here and then go home and, is always the way, a lot of that comes down to time. When I’ve got a short period of time to assess somebody and then treat them, I would rather treat them with the exercise they are going to do at home, rather than teach them something here and then say, and this is how we adapt it for you to carry on at home. It’s silly.’” Therapist (Gilbert et al., 2018) [46];  “One participant was so motivated to practice, that he wanted a device at home. He stated the benefits as being the affordability and the convenience – avoiding problems with traveling to take part in research rehabilitation programs.” Paper author/researcher (Tedesco Triccas et al., 2018) [52];  “There were problems sending information. It would say that it sent it, but it didn’t send the signal because of the area we’re in” (O' Brien Cherry et al., 2017) [43] |
|  | Cost of device is a barrier | “Nine of eleven participants said they would take the ReWalk home if the cost of the machine was not a factor.” Paper author/researcher (Manns et al., 2019) [26];  “Finances were also reported as a potential barrier to being able to travel and access the device.” Paper author/researcher (Cahill et al., 2018) [25];  “…physiotherapists did recognize the potential cost/benefit impact in terms of service efficiency, but the initial purchase price of the robot was seen as quite constraining.” Paper author/researcher (Stephenson & Stephens, 2018) [50];  “The cost was an important consideration not only for patients but also for therapists who could potentially act as gatekeepers to these devices. Thus, a high cost could potentially hinder the utilization of any device for upper extremity rehabilitation.” Paper author/researcher (Elnady et al., 2018) [35];  “You buy an expensive car for the features it has. So, if this has the features that would allow me to walk in a real world like a regular person somewhat, I’d figure out how to buy one.” Patient (Heinemann et al., 2020) [42];  “Replacing face to face contact with patients attending independently to use the device would have some cost implications, particularly in a healthcare system where payment is made for each face to face contact this was identified as a potential problem and barrier to implementation.” Paper author/researcher (Gilbert et al., 2018) [46] |
| Technological challenges | Prior technology experience affects attitudes towards new technologies | “For example, Nick – who enjoys using his computer, phone, camera and playing video games – is content when using different kind of technologies which have positive effects on his behaviour.” Paper author/researcher (Nasr et al., 2015) [57] |
|  | Technical problems with the devices | “Ya’ll got a software problem with the machine . . . the screen would just lock up on me and I’d have to unplug it and then reboot it up” Patient (O' Brien Cherry et al., 2017) [43];  “When you get to level 3 or 4 it would jump on yah . . . it would go up to the next level, just automatically” Patient (O' Brien Cherry et al., 2017) [43];  “Sometimes it took a lot of time to transmit the data and you have to wait too long. You’re all hooked up and everything and you have to sit for ten minutes for the thing to transmit” Patient (O' Brien Cherry et al., 2017) [43] |
| Technological challenges (continued) | Engineering/manufacturing challenges | “’It’s so bulky and when you move it you throw it out of whack, so you have to stay in the same place and work around it’ [Patient]. Most participants reported that they did not move the device from where it was originally set up” Paper author/researcher (O' Brien Cherry et al., 2017) [43];  “… but it’s not really collected any data. That’s because it’s frustrating that the syncing or whatever was supposed to be happening wasn’t occurring or there were recurrent problems. To do it in front of a patient is a little bit embarrassing so I stopped doing it” Therapist (Gilbert et al., 2018) [46];  “They got quite frustrated, one that was quite competitive. She was like, I definitely did it and its saying I didn’t do anything. It said I did really badly” Therapist (Gilbert et al., 2018) [46];  “These comprised of batteries falling off, error signals calling for recalibration of joints, a need to re-program the remote control, and a loosening of a bolt at the hip joint” Therapist (Swank et al., 2020) [44];  “The attach and detach of the system was also criticized as being time-consuming” Paper author/researcher (Eicher et al., 2019) [27];  “three participants felt the assistance provided by the joystick was not noticeable and felt their arms were actively performing most of the movements on their own while playing the games” Paper author/researcher (Sivan et al., 2016) [49];  “… you don’t always want two arms up in the air if you’re trying to reach for something off a shelf” Patient (Kumar & Phillips, 2013) [48];  “It’s important for the controls to be easily accessible because the battery is on your back. If it doesn’t work anymore, if you are stuck somewhere, it’s annoying”; “The battery must last at least an entire day”; “[...] the battery packs [should be] much smaller” Patients (Lajeunesse et al., 2018) [37]; |
| Technological challenges (continued) | Device is cumbersome | “The device took up considerable space in our small kitchen and needs to be smaller than its current size” Patient (Sivan et al., 2016) [49];  “If you don’t walk and if your legs are already heavy, it must be quite difﬁcult to support additional weight” Patient (Lajeunesse et al., 2018) [37] |
|  | Issues with wearing and adjusting the robotic devices | ‘‘The straps got Velcro on both sides but after a couple of weeks of playing the game they started just popping apart, and when I bend down it was kind of uncomfortable and then every time I’d use my foot it’d come apart and then it would tilt the machine a little and then the screen would pop up telling me I tilted the machine’’ Patient (O' Brien Cherry et al., 2017) [43];  “Sometimes, when I feed myself my arm slips out of the sleeve [the sling for the arm supports] because of the angle” Patient (Kumar & Phillips, 2013) [48];  “Such discomfort could be amplified for patients, due to hypersensitivity after a stroke incident” Paper author/researcher (Lo et al., 2020) [56];  “I think the Lokomat was helping me . . . but when it damaged my skin, it was too much for me. It hurt a lot. So that’s why the Lokomat, I think it wasn’t a good thing for me, even if I started again, it wasn’t a good thing for me because it will still damage my skin” Patient (Phelan et al., 2015) [39] |
| Technological challenges (continued) | Other technological challenges | “We put them in the machine and we’re spending a tonne of time just trying to adjust because what we’re measuring isn’t what is getting in the exoskeleton properly” Therapist (Swank et al., 2020b) [29];  “Six participants had problems with joystick calibration/ initializing and required additional training by the research team” Paper author/researcher (Sivan et al., 2016) [49];  “…participants of both groups mentioned that the mechanical resistance, which was installed to motivate the patient to use his or her legs actively, is partly difficult to overcome and physically straining” Paper author/researcher (Eicher et al., 2019) [27];  “An exoskeleton must be high costs, so I guess maybe to have something for the maintenance that doesn’t add additional costs, or to easily have access to services in case of breaking.” Patient (Lajeunesse et al., 2018) [37] |
| Appeal & engagement | Acceptance of devices | “Although nervous about the idea of being in the Lokomat, it seemed that they accepted it as ‘just another thing’ they had to do.” Paper author/researcher (Phelan et al., 2015) [39] |
|  | Robotic devices/interventions are beneficial & fun | “I use it all the time, couldn’t go without it really” Patient (Kumar & Phillips, 2013) [48];  “Because the patients are so positive about it I’ve tended to find a way because one of the biggest challenges we face as therapists is patient adherence” Therapist (Gilbert et al., 2018) [46];  “It’s very good to use technologies for rehabilitation. It’s better/more fun to train with a device, so that’s very good.” Patient (Nasr et al., 2015) [57] |
| Appeal & engagement (continued) | Using novel technologies for rehabilitation | “I trust in new technologies and I really (have) hopes in its help. I would like to be able to use both hands” Patient (Nasr et al., 2015) [57];  “Patients like it because they can see how much they improve […] it’s quite a novel thing, it’s always going to appear attractive.” Therapist (Stephenson & Stephens, 2018) [50] |
|  | Appealing features of the devices/interventions | “It’s also nice that there are not 90 different things, that it is very simple, move forward or move backward or sit down. Simplicity and visual too.” Patient (Lajeunesse et al., 2018) [37];  “It’s been so easy . . . even a dummy like me can understand it. It’s very simplified. Like I said, we don’t even have a computer” Patient (O' Brien Cherry et al., 2017) [43];  “All the therapists were satisfied with the external look and components of the device.” Paper author/researcher (Sivan et al., 2016) [49];  “The robot can achieve a lot more repetitions than we can. (Fiona); Provide something we could never do … repetitions. (Ruth)” Therapists (Stephenson & Stephens, 2018) [50];  “The upper limb devices enabled patients to focus on improving their motor movements, without being impeded by the weight of their weakened arms.” Paper author/researcher (Lo et al., 2020) [56];  “The Ekso gives them more balance so they’re not focusing so much on their balance. They can focus more on the gait aid … instead of having to put so much focus into where their feet are that they can’t even think about moving the walker ahead or moving the cane.” Therapist (Read et al., 2020) [40];  “I liked the concept of the levels progressively becoming harder.” Patient (Sivan et al., 2016) [49];  “The system provided me with stability during walking, I experienced the provided support by the system for my disabled leg as very positive” Patient (Eicher et al., 2019) [27];  “The therapists liked the idea of being able to adjust the assistance to the system based on individual deficits. […] also appreciated the concept of the assistance adjusting automatically based on individual performance.” Paper author/researcher (Sivan et al., 2016) [49];  “….but when Ekso is done right, I really don’t work hard at all. . . generally you’re just breezing. . . it’s like it’s doing the work for you. So, it’s just so much less wear and tear on us as well.” Therapist (Swank et al., 2020) [44] |
| Appeal & engagement (continued) | Aspects of the devices/interventions the participants did not like | “[…] off the bat, it sure looks like a bionic man or woman. . . It’s a bit strange” Patient (Lajeunesse et al., 2018) [37];  “I wish the exoskeleton was made of ﬁber, so that it looks the most like the human body” Patient (Lajeunesse et al., 2018) [37];  “I got bored using the device. Going to the gym suits me more as I meet other friends and do a variety of exercises” Patient (Sivan et al., 2016) [49];  “The Lokomat does all the work and in walk[ing] you do the work. [….] But in physiotherapy, it’s fun and you’re doing all of the work, so that . . . that’s the thing that really makes me happy about physiotherapy. Like, you don’t just stand there and some . . . something else or somebody else does all the work for you” Patient (Phelan et al., 2015) [39];  “Several participants talked about the effort required of the arms, “my arms getting used to it. ‘Cause obviously I wouldn’t be using those muscles as much, just wheeling’.” Patient (Manns et al., 2019) [26];  “Standing up was described as “sometimes rather brutal” and they were “forced into standing”. Once the standing procedure started, they were unable to stop or reverse it, even if they experienced pain or a sudden increase in spasticity.” Paper author/researcher (Thomassen et al., 2019) [45];  “Ekso was described as a massive device that could feel  rather tight and unnatural to wear. The participants described that they felt completely locked in it.” Paper author/researcher (Thomassen et al., 2019) [45];  “Walking on my own. Just ‘cause, like, there’s a lot of stuff attached to the Lokomat. So I kind of been just like . . . ‘I’m free . . .’ ([Laughs]) ‘I can finally just normally walk’.” Patient (Phelan et al., 2015) [39];  “There was some of the games, you could say were a bit tedious. That was maybe because they didn’t tax you enough” Patient (Sweeney et al., 2020) [51];  “The participants expressed that they felt that the computer graphics of the games during RT were not accurate, nor well designed resulting in feelings of confusion.” Paper author/researcher (Tedesco Triccas et al., 2018) [52];  “David believed that people with stroke would not like the idea of graphs and bar charts as a means of receiving feedback. ‘The person with stroke will realise when they progress by looking at the function of their hands and they gain the sense of improvement through experience by doing the exercises and observing the progress’” Patient (Nasr et al., 2015) [57];  “If I’m only able to do 10 minutes of gait training in a robot, and I can get 35 minutes in on a treadmill, I think there is a dose difference there.” Clinician (Heinemann et al., 2018) [28];  “Pat felt that the exoskeleton acted as a kind of barrier “between me and the patient. With the parallel bars, it is me and the patient. … That is a tool I am in control of. I have no sense of control of the exo[skeleton].” Pat’s comments highlight that, for a few participants, the exoskeleton felt disembodied and robotic.” Therapist & Paper author/researcher (Mortenson et al., 2020) [38] |
| Appeal & engagement (continued) | Uncertainty & cautiousness | “It’s not that I’m less enthusiastic, it’s just that I know from my experience the practicalities of it and the realistic expectations that the technology can and cannot do. So if the technology is evolved to the point where it would be practical for my needs then great, but it’s just not there yet.” Patient (Heinemann et al., 2020) [42];  “Essentially we’re going to need to see with our own eyes that it’s actually helping people because even if we’re trained, even if we feel comfortable in its use but we don’t value its use, we’re not going to use it.” Therapist (Flynn et al., 2019) [53];  “Some participants were unsure about how it fit with current practice, given the overall complexity of the technology, as Mel related: It’s more functional to be on a treadmill practicing your walking around rather than having this iron man suit strapped to you …” Paper author/researcher & Therapist (Mortenson et al., 2020) [38] |
|  | Motivating factors for patients to use the device or intervention | “Cause, God, if I could just walk again . . . You don’t know . . . how much it would mean to me” Patient (Danzl et al., 2013) [41];  “I couldn’t motivate myself the same [at home] as I could up here. You need that wee bit of push” Patient (Sweeney et al., 2020) [51];  “Continued engagement was perceived to be linked to tailored feedback” Paper author/researcher (Stephenson & Stephens, 2018) [50];  “He emphasised the significance of having a personalised human interaction with the user to motivate them to perform the therapeutic games. For example, seeing a therapist talking to the user and instructing them during the programme could be encouraging for them, the presence of a human agent either a therapist or a mentor could be uplifting for the user” Paper author/researcher (Nasr et al., 2015) [57]  “You were competing against yourself, eh. and driving yourself on to get the beneﬁts out of it” Patient (Sweeney et al., 2020) [51];  “I’m always interested in things that are outside the box. To me, that’s innovative thinking . . . I think it’s better to ﬁnd the root of the problem and try to ﬁx it instead of takin’ a pill to just decrease the symptoms” Patient (Danzl et al., 2013) [41] |
| Appeal & engagement (continued) | What motivated clinicians to recommend use of the device/intervention | “. . . that speaks volumes to motivating them to do a lot of their other functional tasks, because some of the other goals they might not want to work on, but we can kind of use it as like a “carrot” and be like: ‘All right, if you do this transfer, and work on this transfer this day, then by the end of the week we will be able to try to get you up on the robot’ . . . . I think that has a big ... inﬂuence on a lot of other stuff we try to do.” Clinician (Heinemann et al., 2018) [28];  “Clinicians reported they would actively try to empower patients to use the device as part of their normal rehabilitation to promote patient compliance and release clinician time and in these situations MUJO [the robotic device] was effective” Paper author/researcher (Gilbert et al., 2018) [46] |
| Appeal & engagement (continued) | Facilitators to use of the devices/interventions | “So yeah, also the timing of getting the arm support was I think perfect, . . . at that moment where I still had enough mobility to kind of do a few things myself, and kind of get used to the arm support before it’s kind of too difficult to get used to something like that” Patient (Kumar & Phillips, 2013) [48];  “So I built it into my regime every day. I’d go to the machine first thing in the morning before anyone was using it, do about half an hour on it and then just crack on for the rest of the day. So I think it sped up my recovery considerably” Patient (Gilbert et al., 2018) [46];  “I wouldn’t want to get a programme from someone at the gym to show me how to use my shoulder. That’s because I am in rehab. Having been to the gym and having had shoulder surgery and they have suggested rowing, that was a personal trainer, that is not the level of person I would expect to be telling me – setting a programme for rehabilitation” Patient (Gilbert et al., 2018) [46] |
| Supportive interactions & relationships | Therapist-patient relationship is important | “…he wants to see you working … he doesn't want to see you dilly dallying or dossing […] he wants a hundred percent of whatever your hundred percent is” Patient (Cahill et al., 2018) [25];  “It’s like when they, like, really listen, and when I need something adjusted that they adjust it and that . . . I don’t really like going if I’ve had some bad sessions where they weren’t really listening or something. And I find that, like, [one therapist], like, she really listens and I don’t really have that issue . . . but then with [the other therapist] I think we have a bit more of that issue. I’m not really sure why that is, it just might be because she focuses more on, like, the physical aspect of things, so yeah . . . I feel like I’m a bit more connected to them [one therapist and assistant] rather than I am with [other therapist and assistant]” Patient (Phelan et al., 2015) [39];  “If she doesn't enjoy having an interaction with the therapist, then it's not going to work... no matter how good the equipment is. . . it's not just the machine, it was the therapist and the assistant.” Parent (Beveridge et al., 2015) [34];  “You know, what I would like to say about the Lokomat, I think they’re [therapy team] doing a good job. And they’re not . . . they’re not pressuring you to . . . it’s like they care about you, like . . . if you’re hurt or . . . That’s what I like about them, is that they care about people.” Patient (Phelan et al., 2015) [39] |
| Supportive interactions & relationships (continued) | Managing patients' expectations | “I thought it would help with all sorts, different kind of stuff, I thought I would be able to pick stuff up and things, and pick bottles of pop up and stuff, but when I got it I wouldn’t be able to pick heavy stuff up. But I thought that would do that but obviously it didn’t” Patient (Kumar & Phillips, 2013) [48];  “The other thing that I think is really critical are patient expectations. I think in this room we are all 100 percent, it’s about the patient, but we also have to be open and honest with them about how they can really use these systems, and unless they come and try it, they won’t know.” (Heinemann et al., 2018) [28];  “D2, for example, explained that she had been more realistic and open about patients’ prospects of walking when she started working at the hospital, but that over time she began to think that it was better to ‘leave things to time’. In her words, ‘they are not fond of doctors who tell the truth’. As D1 and D5 also explained: Even the fact that they see themselves standing up – the perception to be normal – has a very positive impact. It is very helpful in terms of motivation and one’s psychological state of mind. [But it is true that the Lokomat presents] very low levels of evidence in terms of functionality. Do we openly admit that? It is not very good to tell patients everything [at the outset]. Hope is very important for them not to sink into depression. They do not want to hear it. (D1)” Paper author/researcher & Doctors (Bezmez & Yardimci, 2016) [54] |
| Supportive interactions & relationships (continued) | Support from family is beneficial | “You know when my brother comes in I show off, it makes me show off ‘cause he’s seen what I was like when I first come out of hospital, I couldn’t get out of this wheelchair and when I walking again a bit better, course I’ve nearly fallen over like but that’s beside the point, I’m motivated knowing…There’s nothing more good in my opinion for a stroke victim is to know people care” Patient (Nasr et al., 2015) [57];  “Sam’s father expressed: ‘It’s more important for kids that they get motivated. And I think that [Sam] values a lot what I tell him, and he always wants me to be happy’. Mark, when completing the facial expression on his image-captioning activity, indicated:  Interviewer: And tell me about the face you drew, it looks like a smiley face?  Mark: Yeah. I’m smiling at her [mother] because she always makes me smile.” Parent & Patient (Phelan et al., 2015) [39];  “The walking pattern is very important. .. Because Sam could walk ..... by himself. But not correctly. So the best and correct walking style is very important for Sam and us.” Parent (Beveridge et al., 2015) [34] |
| Benefits for physical, psychological, and social function(ing) | Physical benefits of using the device | “Participants reported that following every session, they felt that the intervention resulted in an improvement in their daily tasks at home outside of the treatment sessions, such as opening doors, gardening, dressing, and that these positively affected their quality of life and increased their confidence.” Paper author/researcher (Tedesco Triccas et al., 2018) [52];  “I am zipping up my coat using both hands, which I could not do before.” Patient (Sivan et al., 2016) [49];  “I could help my wife to do small domestic jobs outside and I could take care of my things a little bit more.” Patient (Lajeunesse et al., 2018) [37];  “My gait improved after 2 to 3 training sessions, unfortunately the number of training sessions was not enough, otherwise gait would have improved a lot more” Patient (Eicher et al., 2019) [27];  “Addressing walking and what was termed "correct" walking was seen by parents as a means of affecting other areas of their children's lives such as decreasing falls and achieving independence.” Paper author/researcher (Beveridge et al., 2015) [34];  “Now I can dress myself, cut my food, and get in and out of the bed. I can actually just stand and grab this and be on my way instead of waiting for somebody to help me” Patient (O' Brien Cherry et al., 2017) [43];  “I could use it as an assessment tool to get specific markers to begin with. They go off and do their home exercises in any other ways and then come back and then I could reassess using it and see what happens numbers-wise with the [Patient] App” Therapist (Gilbert et al., 2018) [46] |
| Benefits for physical, psychological, and social function(ing) (continued) | Psychological benefits of using the device | “After he’d been cut off from his physical therapy last November, he got depressed and he would get angry with me when I would make him do his exercises. Now he’s in a much better mood and his depression has lifted” Carer (O' Brien Cherry et al., 2017) [43];  “….all participants reported a sense of achievement following exoskeleton walking and in particular the psychological beneﬁt of standing upright ‘looking people in the eyes, rather than the waist’ and moving in a walking motion; a subject deemed emotive in interviewer ﬁeld notes.” Paper author/researcher (Cahill et al., 2018) [25];  “She goes ‘Mom, look, I can keep up with my brothers now.’ And she credited the Lokomat to that. And she was proud of herself that she could keep up with her brothers and walk as fast as her brothers . . . And that was important to her. And she could keep up with me. That was important to her. So yeah, for her confidence level and her self-esteem, 100% it’s boosted it.” Parent (Phelan et al., 2015) [39];  “The ability that some feel that they can stand up [. . .] and here they are up walking, and they are like: ‘Can you videotape this? Can you video it?’ So, I think ... psychosocial[ly] ... this gives them that hope that . . . ‘Someday I may be able to walk again.’” Clinician (Heinemann et al., 2018) [28];  “Another stated, ‘to be able to go out in the sun and go for a walk – it’s unbelievable what that does for you’.” Patient (Manns et al., 2019) [26];  “The device gave me a sense of purpose in life, something I looked forward to every day when I got up.” Patient (Sivan et al., 2016) [49];  “One stated, ‘It was hard at first because my mind is broken and the leg don’t work unless the mind is working’. He later stated, ‘. . . the game made me realize that my leg was there’. The games reportedly improved the memory of their arm or leg without users being explicitly conscious of the process.” Paper author/researcher (O' Brien Cherry et al., 2017) [43] |
| Benefits for physical, psychological, and social function(ing) (continued) | Social benefits of using the device | “You are then suddenly ﬁtter and more able … to even just interact with other people because rather than being out breathless, or struggling, you are more able to get out, get around” Patient (Cahill et al., 2018) [25];  “Feeling more engaged in social situations rather than . . . being in a chair looking up and you can’t really hear what people are saying in a crowded room.” Therapist (Heinemann et al., 2018) [28] |
| Benefits for physical, psychological, and social function(ing) (continued) | Psychological issues due to the device or intervention | “I felt a bit insecure in it because some of, like, adjustments had to be made and it still wasn’t totally perfected even like without me in it, like, it still wasn’t perfect and we knew that. So I felt a little bit like freaked out to be in it, but when they were like, you know, talking to me and, like, reassuring me a bit more, I felt more comfortable…” Patient (Phelan et al., 2015) [39];  “I was just a bit embarrassed because it stuck out a bit, and it’s a bit, I just weren’t very happy using it” Patient (Kumar & Phillips, 2013) [48];  “In some cases, participants felt the research reminded them that they still have to deal with their impairment.” Paper author/researcher (Tedesco Triccas et al., 2018) [52];  “In reflecting on stopping training, they further stated, ‘it feels like I’m getting fired or something’.” Patient (Manns et al., 2019) [26];  “When I came home back [released from the hospital] my kid brought me a wheelchair,...I said never, I would get into one of these,...I am walking around for 6 months now.” Patient (Elnady et al., 2018) [35];  “The way robotic training was organized affected how some of therapists at this hospital saw themselves professionally. Some therapists did not have a sense of patient ownership and felt that they were no longer clinicians. ‘You become a technician and we’re not technicians, we’re clinicians who use our clinical reasoning’.” Therapist (Lo et al., 2020) [56];  “They described the training sessions and the subsequent therapy sessions as an unspeciﬁed workload of “a lot,”“a lot of information,”“tiring,” or “challenging,” indicating that a great deal of multifaceted mental work was put into functioning as an Ekso expert and physiotherapist.” Paper author/researcher (Read et al., 2020) [40] |
| Benefits for physical, psychological, and social function(ing) (continued) | Some participants had mixed experiences | “While on the robot can achieve good movements … not sure how much that carries over when not strapped to the robot.” Therapist (Stephenson & Stephens, 2018) [50];  “The system seems rather suitable for more severely affected people” Patient (Eicher et al., 2019) [27] |
|  | No improvement from using devices or intervention | “The remaining 12 participants did not notice any improvement in arm power and ability.” Paper author/researcher (Sivan et al., 2016) [49] |
| Expanding and sustaining therapeutic options | Limited or absent pathways for transitioning from the medical model to that of a wellness approach following early injury rehabilitation | “Participant B aptly summarised the theme of re-integration and  wellness stating: ‘you feel more integrated into normal society rather than being again a clinical spinal injured person being worked on by professional […] in a hospital environment. You're back into society and working out with everyone else.’” Patient (Cahill et al., 2018) [25] |
|  | Implementation & contextual factors | “Participants indicated that the ad-hoc implementation of robotics was leading to suboptimal rehabilitation.” Paper author/researcher (Lo et al., 2020) [56];  “The other thing we’re fortunate here I think there is quite good support from line managers.” Therapist (Flynn et al., 2019) [53];  “Location and space considerations were often overlooked in the implementation but yet had an impact on the organization and delivery (scheduling) of care.” Paper author/researcher (Lo et al., 2020) [56] |
|  | Training | “it’s very challenging for anybody to get funding to purchase one, and then to….ﬁnd places where therapists are trained on how to use them….” (Heinemann et al., 2018) [28];  “…pre-reading would have been helpful [so that future trainees] … know what they’re getting into and to know that they have the aptitude of passing.” Therapist (Mortenson et al., 2020) [38] |
| Expanding and sustaining therapeutic options (continued) | Time management & resources | “I do feel I am quite negative about them … I understand the theory, I wish we had more time to use them.” Therapist (Stephenson & Stephens, 2018) [50] |
|  | Having appropriate staff | “Patient-by-patient basis … there should be a physio(therapy) assessment to lead into the treatment for an assistant to take over.” Therapist (Stephenson & Stephens, 2018) [50] |
|  | Costs | “It’s expensive to produce, but I think it is something that should normally be provided by the health program.” Patient (Lajeunesse et al., 2018) [37];  “If you’re looking at cost-effectiveness you can treat two patients at the same time with one physio(therapist).” Therapist (Stephenson & Stephens, 2018) [50] |
|  | Accessibility & funding | “I think in theory it’s a good piece of equipment, but it just needs – it’s not the equipment that needs to be developed so much, its more the availability of it” Therapist (Gilbert et al., 2018) [46];  “I think without [...] extended benefits, without insurance companies maybe buying into that, I think [only] relatively small grouping of clients would be able to afford it.” Therapist (Elnady et al., 2018) [35];  “Participant 3 related how they gave up waiting for funding to come through and purchased the arm supports privately: “It would have taken too long so we just decided to buy them” Patient (Kumar & Phillips, 2013) [48] |
|  | Patient suitability & screening | “I think out in the community people are going to fall […] I think there is risks for injury, for fracture, for . . . bone fractures or skin injury. But I think the key to that in reducing that is proper screening and the training that goes behind it the screening process and the education is really important to reduce those risks” Clinician (Heinemann et al., 2018) [28];  “Yeah, either they have a foley or they’re doing intermittent cathing and have a bowel program. . . . I’ve had some issues with that, but never – they usually have a brief on them. . . I have had to get somebody out because they’re like, ‘I feel like I need to go to the bathroom’. But, I haven’t had any episodes of incontinence which is surprising, but good.” Therapist (Swank et al., 2020) [44];  “In addition to having little or no visual impairment Jamie recounted: To be eligible you have to be under 200lbs or so. … You have to have three [limbs] that are functional and that are able to have sufficient strength. You have to have sufficient hip [and knee] range of motion, … They have to have some good thorax or core, they also have to obviously have been doing some standing and walking already to be able to have the endurance to tolerate it.” Therapist (Mortenson et al., 2020) [38];  “It’s becomes not that effective because what we call ‘fighting with it’. As I said, the machine moves in this pattern, in this time, and there’s no change to that. If they don’t join in with that, it’s not helping their therapies.... We had a few that we actually had to say: ‘You know what, this isn’t the right therapy for them’ because of that reason.” Therapist (Lo et al., 2020) [56];  “It (RT-UL device) was only really appropriate for a few patients.” Therapist (Flynn et al., 2019) [53];  “From the interviews, we found that robotics seemed to be the preferred treatment option for patients with severe impairments.” Paper author/researcher (Lo et al., 2020) [56];  “Others thought that robotic exoskeletons would be useful as a therapy tool for individuals who have some ability to walk. For example, I think anybody new, like unfortunately anybody who’s newly injured, it would give them a big boost, just hope and mentally, spiritually, psychologically. I think that just seeing something like that would give them the push and the drive to strengthen what they can train and hopefully build up to wanting to use a machine like that. Because that’s the ultimate goal. At least in my mind. Anybody who’s paralyzed, you want to be able to walk again. So just seeing that is like, ‘I can get to that level,’ or I can get to that hopefully it could be a goal for them. Whether it’s realistic or not, it’s realistic in your mind.” Patient (Heinemann et al., 2020) [42];  “I tried to get one, but I got disqualified because I have HO [heterotopic ossification] in my left leg. Yeah that’s what I’ve understood is that it’s not really suitable for quads. It’s more of a paraplegic application.” Patient (Heinemann et al., 2020) [42];  “This affected which patients had access to the robotic training. Instead of a needs-based approach, patients who could afford the more expensive robotic therapy had access to it.” Paper author/researcher (Lo et al., 2020) [56] |
| Expanding and sustaining therapeutic options (continued) | Research can be limited in terms of day-to-day relevance | “Whether or not that is sustainable actually outside of a study, within the NHS would be another matter … modify what happens in a clinical trial onto the ward.” Therapist (Stephenson & Stephens, 2018) [50] |
|  | What should such devices help you achieve/activities you should be able to do with a robotic device | “….the needs were different; participants with stroke were in the favor of assistive devices, whereas therapists demonstrated a preference for therapeutic devices.” Paper author/researcher (Elnady et al., 2018) [35];  “Help with ﬁngers at the same time (if ﬁngers don’t work you can’t use your arm” Patient (Hughes et al., 2011) [47];  “Ideally, one would expect to work on hand opening and closing while performing reaching movements. The concept of bimanual involvement is good to see.” Therapist (Sivan et al., 2016) [49];  “think there would be pros to getting the distal because maybe if they [patients]start to activate their hand more, then we’ll actually see better proximal, I think that the research shows we don’t just lift our arms for the sake of lifting our arm[s], I mean you’re always moving your upper limbs for a purpose.” Therapist (Elnady et al., 2018) [35];  “They described general goals like being able to improve hand function, doing daily activities and reaching, grasping and holding objects” Paper author/researcher (Nasr et al., 2015) [57];  “The walking pattern is very important. .. Because Sam could walk ..... by himself. But not correctly. So the best and correct walking style is very important for Sam and us.” Parent (Beveridge et al., 2015) [34];  “It would be of great help if it allowed me to walk longer distances. At a certain point, I become tired.” Patient (Lajeunesse et al., 2018) [37];  “To clean up, to clean the ceiling, to use the vacuum cleaner.” Patient (Lajeunesse et al., 2018) [37];  “I am not capable of mowing the lawn because I don’t have the required strength. But, with something that I could remain standing, I could at least cut it slowly with the grass trimmer.” Patient (Lajeunesse et al., 2018) [37];  “To go walk the dog, to play with the dog, just that.” Patient (Lajeunesse et al., 2018) [37];  “To be able to walk with someone, with my wife, next to her” Patient (Lajeunesse et al., 2018) [37];  “To go to festivals, to be able to remain standing, to walk around, to move” Patient (Lajeunesse et al., 2018) [37] |
| Expanding and sustaining therapeutic options (continued) | Appropriate settings | “Constraints are put back onto you. You're just made […] to feel disabled. Whereas at just a regular gym where there's lots of able bodied people working out as well, you feel just like anybody else, normal” Patient (Cahill et al., 2018) [25];  “Two participants commented they would be more comfortable using it with people who had similar disabilities rather than playing in front of non-disabled people.” Paper author/researcher (Sivan et al., 2016) [49];  “If it’s needed, we really like to train at home.” Patient (Nasr et al., 2015) [57];  “Ideally for this … would be to have an exoskeleton in the [rehabilitation hospital]. Everybody gets to use it. Everybody gets to be comfortable in it in this clinical setting, with the physios, who know the EKSO, know the patient. Then, afterwards […] ideally there would be an Exoskeleton in their area, in a gym.” Patient (Cahill et al., 2018) [25] |
|  | Importance of tailoring devices | “….when the hand device is tailored to the size of the hand of an individual user it feels comfortable.” (Ates et al., 2014) [58];  “Everyone has their own situation...We’re talking about different things for different situations...there isn’t one size fits all.” Patient (Elnady et al., 2018) [35] |
|  | Important to maintain human presence | “An important aspect that was pointed out by many participants was their concerns about the lack of interaction from a real therapist having the system at home. Steven expressed his concerns in the following excerpt: There seems to be so little out there and I’m worried that people are going to feel more isolated” Patient (Nasr et al., 2015) [57];  “You need to be there with your patient … at all times when they are on the robot … looking at their posture, concentration … reminders about sitting upright … safe in the chair.” Therapist (Stephenson & Stephens, 2018) [50] |
| Expanding and sustaining therapeutic options (continued) | Independent use of robotic devices | “A participant said, ‘The device is better because it ain’t talking back. The machine just do what it do and I can focus on the machine and trying to beat it’.”Patient (O' Brien Cherry et al., 2017) [43];  “Some participants also felt that using the device reduced frustrations that occurred when their caregiver, often their wife, would act the role of therapy coach. One caregiver said, ‘He didn’t like me running him through all of the exercises’.” Carer (O' Brien Cherry et al., 2017) [43];  “Besides, it’s important to use the device by yourself (put the device on and off), without help from someone else to be more independent.” Patient (Nasr et al., 2015) [57] |
|  | Devices seen as complimentary to traditional therapy | “It has to be an extra to therapy … I never saw it as a main avenue of treatment.” Therapist (Stephenson & Stephens, 2018) [50];  “That is why we’ve been doing a combo so the patients get a lot of repetition in with body weight supported treadmill training, and then with Ekso the patients can remember what we’ve been talking about and then they can take over” Therapist (Swank et al., 2020b) [29] |
|  | Comparisons with treadmills and wheelchairs | “With treadmill, you get tons of repetition so we take them there all the time.” Therapist (Swank et al., 2020b) [29];  “Although the use of Ekso evoked positive feelings, none of the participants would trade the wheelchair, as it is far superior to Ekso in everyday life.” Paper author/researcher (Thomassen et al., 2019) [45] |
|  | Training goals | “Therapists stated that training should be oriented at a patient’s goal(s) and his/her ability to accomplish these goal(s).” Paper author/researcher (Hochstenbach-Waelen & Seelen, 2012) [55];  “Therapists reported that training should be motivating  and challenging for patients; inclusion of a gaming element can contribute to both requirements.” Paper author/researcher (Hochstenbach-Waelen & Seelen, 2012) [55];  “This lack of challenge was further perceived as placing limitations on their potential....” Paper author/researcher (Cahill et al., 2018) [25] |
| Expanding and sustaining therapeutic options (continued) | Therapist training | “Despite the challenging technical aspects of the training, the majority of therapists were positive about their overall training experience; for example, Les noted: ‘there was a lot to learn …but the training was adequate to get us confident that we could be safe with the device’.” Paper author/researcher & Therapist (Mortenson et al., 2020) [38];  “.. when the people from the different companies they come, they tell you how to use the devices but very general way. They mainly tell you the safety information but they are not really clinicians. Normally they don’t know too much about therapy. At the end you have to find yourself the way to use the devices with different patients.” Therapist (Lo et al., 2020) [56] |
|  | Design-related suggestions | “It’s important for the controls to be easily accessible because the battery is on your back. If it doesn’t work anymore, if you are stuck somewhere, it’s annoying.” Patient (Lajeunesse et al., 2018) [37];  “The device should be installed ‘ready-to-go’. This implies that:  a. it should be able to start immediately, without excessive start-up procedures; b. it should be easy to operate; c. it should preferably be portable. In this way, loss of individual therapy time is minimized.” Paper author/researcher (Hochstenbach-Waelen & Seelen, 2012) [55];  “I don’t want something too big, because if it is bulky, you cannot do a lot of things with that.” Patient (Lajeunesse et al., 2018) [37];  “If the battery dies and you are in a movement, what will happen? Do you have an emergency battery? Is there anything that indicates you that the battery is about to stop, that you must recharge it?” Patient (Lajeunesse et al., 2018) [37];  “David suggested that users should be able to simply connect the system to their own TV and play the games. He was concerned that the current system would be in the way when they have visitors especially when their grandchildren visit them.” Paper author/researcher (Nasr et al., 2015) [57];  “One patient said that the leg shells ‘do not fit and therefore individual lower leg shells would be of benefit’.” Patient (Eicher et al., 2019) [27];  “Could the handle incorporate pronation/supination and finger movements to work on distal muscles as well?” Therapist (Sivan et al., 2016) [49];  “Brain activity would be cool, actually having them [patients] like think about the movement; it seems smart for somebody who had a stroke, which would be a good idea. EMG [electromyography] maybe if they’re showing small amounts of muscle activity, so then they [patients] might then back learn the pattern more, and with that help for further activation.” Therapist (Elnady et al., 2018) [35] |
| Expanding and sustaining therapeutic options (continued) | Personalising the devices or intervention | “They should be challenging, so not too difficult but also not too easy that they become boring.” Paper author/researcher (Nasr et al., 2015) [57];  “…For example, you know get some music to match what the user is doing, it’s all motivation, and the simpler the better…” Patient (Nasr et al., 2015) [57];  “I liked the puzzle and chase game in particular as the higher levels were interesting and complex.” Patient (Sivan et al., 2016) [49];  “It should be as rounded as possible and should ﬁt as much as possible the user’s skeleton or appearance. That would be perfect.” Patient (Lajeunesse et al., 2018) [37];  “Apart from different physical capabilities, the participants also  had different levels of knowledge about computers, which has to be taken into account when designing the system.” Paper author/researcher (Nasr et al., 2015) [57] |
| Expanding and sustaining therapeutic options (continued) | Other suggestions to increase uptake and engagement with devices | “My gait improved after 2 to 3 training sessions, unfortunately the number of training sessions was not enough, otherwise gait would have improved a lot more” Patient (Eicher et al., 2019) [27];  “Therapists reported that the system should be able to give clear instructions to the patient about the exercise or task to be performed in a variety of ways, like an instruction movie, or verbal or written instructions.” Paper author/researcher (Hochstenbach-Waelen & Seelen, 2012) [55];  “The system provides the user with an opportunity to communicate with their therapist by sending a text message. This was problematic for Steven because after his stroke he does not leave space between the words and that could be confusing for the recipient of the text message. He suggested that sending an audio or video message to the therapist would be easier for him.” Paper author/researcher (Nasr et al., 2015) [57]; |
|  | Devices providing feedback are useful & desirable | “I think it’s really fun to be on the Lokomat, because you see in that screen, like, the screen can tell you what you’re doing, not just like when you’re walking and you don’t know how you’re doing, because, well . . . there’s no feedback.” Patient (Phelan et al., 2015) [39];  “…the visual feedback of performance should be clearer and easier to interpret” Paper author/researcher (Tedesco Triccas et al., 2018) [52];  “While some participants seemed to be motivated with scores as a means of receiving feedback on their progress, others believed that effective feedback may take more than one form. Users of different backgrounds can become motivated when they receive meaningful and culturally-responsive feedback.” Paper author/researcher (Nasr et al., 2015) [57];  “Biofeedback was seen as a desired feature by some therapists to allow therapists and their patients to have information relating to joint position, muscle use and activation.” Paper author/researcher (Huq et al., 2012) [36] |
| Expanding and sustaining therapeutic options (continued) | Other recommendations | “I’ve noticed that there are a lot of treatments that are utilized, especially by the military, and they’re usually tested on men. And so when they get a female patient it seems to throw everybody off. Nobody knows what to do. Nobody sure how to fit it. Nobody sure how it’s going to react. Nobody knows anything about how it will relate to a female patient.” Patient (Heinemann et al., 2020) [42];  “Because you will lose your physio skill as well, if you’re just on the robot the whole time. You still need to be able to do some sort of clinical work, so that you’re not losing skill and you want to continue to upskill as well.” Therapist (Lo et al., 2020) [56] |
